# Filling Predictable and Unpredictable Gaps, with and without Similarity-Based Interference: Evidence for LIFG Effects of Dependency Processing

**DOI:** 10.3389/fpsyg.2015.01739

**Published:** 2015-11-16

**Authors:** Kimberly Leiken, Brian McElree, Liina Pylkkänen

**Affiliations:** ^1^Department of Linguistics, New York University, New YorkNY, USA; ^2^Division of Neurology, MEG Center, Cincinnati Children’s Hospital Medical Center, CincinnatiOH, USA; ^3^Department of Psychology, New York University, New YorkNY, USA; ^4^NYUAD Institute, New York University Abu DhabiAbu Dhabi, UAE

**Keywords:** neurolinguistics, left inferior frontal gyrus, magnetoencephalography, Filler-gap dependency, object relative clause, verb phrase ellipsis, right node raising, similarity-based interference

## Abstract

One of the most replicated findings in neurolinguistic literature on syntax is the increase of hemodynamic activity in the left inferior frontal gyrus (LIFG) in response to object relative (OR) clauses compared to subject relative clauses. However, behavioral studies have shown that ORs are primarily only costly when similarity-based interference is involved and recently, Leiken and Pylkkänen (2014) showed with magnetoencephalography (MEG) that an LIFG increase at an OR gap is also dependent on such interference. However, since ORs always involve a cue indicating an upcoming dependency formation, OR dependencies could be processed already prior to the gap-site and thus show no sheer dependency effects at the gap itself. To investigate the role of gap predictability in LIFG dependency effects, this MEG study compared ORs to verb phrase ellipsis (VPE), which was used as an example of a non-predictable dependency. Additionally, we explored LIFG sensitivity to filler-gap order by including right node raising structures, in which the order of filler and gap is reverse to that of ORs and VPE. Half of the stimuli invoked similarity-based interference and half did not. Our results demonstrate that LIFG effects of dependency can be elicited regardless of whether the dependency is predictable, the stimulus materials evoke similarity-based interference, or the filler precedes the gap. Thus, contrary to our own prior data, the current findings suggest a highly general role for the LIFG in dependency interpretation that is not limited to environments involving similarity-based interference. Additionally, the millisecond time-resolution of MEG allowed for a detailed characterization of the temporal profiles of LIFG dependency effects across our three constructions, revealing that the timing of these effects is somewhat construction-specific.

## Introduction

A classic finding within the cognitive neuroscience of language processing is that the comprehension of object relative (OR) clauses, such as (1), have been found to engender more hemodynamic activity in the left inferior frontal gyrus (LIFG, aka “Broca’s Area”) than subject relative (SR) clauses, such as (2) (e.g., Just et al., 1996; Stromswold et al., 1996; Caplan et al., 2000, 2008; Keller et al., 2001; Constable et al., 2004). This observed difference in hemodynamic activity mirrors the behavioral findings that ORs are more costly to process than SRs by various measures (Holmes, 1973; Hakes et al., 1976; Wanner and Maratsos, 1978; Holmes and O’Regan, 1981; Ford, 1983; Waters et al., 1987; King and Just, 1991).

(1) the fireman [(who_i_) the deputy called *t*_i_] saved the sailor
(2) the fireman [who_i_ *t*_i_ called the deputy] saved the sailor

In tandem with hypotheses developed from an older body of aphasia studies, this effect has given rise to the popular conception that Broca’s area is somehow linked to syntactic processing (Berndt and Caramazza, 1980; Damasio and Damasio, 1989; Zurif, 1995; Grodzinsky, 2000). Of specific proposals, the narrowest in terms of LIFG function hypothesizes that this region is specifically responsible for the processing of displacement or movement (Grodzinsky, 2000; Ben-Shachar et al., 2003; Grodzinsky and Santi, 2008). It is important to note that these theories have primarily been tested using relative clause structures, such as those described above, which contain movement operations (or “transformations”) that result in a long-distance dependency between two elements. Therefore, it is unclear whether it is the movement process itself or the consequential relation between non-adjacent components that induces the increase in activation. A more general set of hypotheses, but still specific to linguistic processing, includes the “linearization” computation (Bornkessel et al., 2005; Grewe et al., 2005), and the process of “unification” (Hagoort, 2003, 2005). Linearization involves maintaining hierarchical orderings of the members of a linguistic dependency. If this process takes place in the LIFG, then a violation of linguistic hierarchy should yield increased LIFG activity. Therefore, as English-type languages show a preference for subjects to precede objects, the LIFG effect for ORs could be taken to reflect the reversal of the subject–object order. Unification, on the other hand, is the process of integrating lexical information from a single word into a larger syntactic frame that has been retrieved from memory. Therefore, if this computation takes place in the LIFG, then integration of individual lexical items into the OR syntactic frame retrieved from memory might generate increased LIFG activity.

The above proposals contrast with hypotheses linking the LIFG primarily to non-language-specific processes, such as working memory (Caplan et al., 2000, 2008; Fiebach et al., 2001, 2005; Kaan and Swaab, 2002; Rogalsky et al., 2008; Makuuchi et al., 2009) or cognitive control (Botvinick et al., 2001; Miller and Cohen, 2001; Novick et al., 2005; Braver et al., 2007). Under both of these types of accounts, the LIFG increase relates not to any language-specific structural operation, but rather to the fact that, in ORs (but not in SRs), two noun phrases (NPs) are encountered prior to the verb, taxing working memory and/or inducing conflict.

The goal of the present work was to contribute to our understanding of LIFG function in language processing by examining LIFG dependency effects with a different methodo logy and a broader range of dependencies than previously studied, as well as by manipulating variables that affect memory retrieval operations in resolving a dependency. We employed magnetoencephalography (MEG), which, contrary to the traditional hemodynamic methods, allowed for a detailed temporal characterization of LIFG activity. Our design systematically varied not only the presence of dependency structures, but also the extent to which dependent structures elicited retrieval interference. In addition to commonly studied object extractions, we also explored dependencies resulting from verb-phrase ellipsis and right-node raising. Contrasting these constructions with ORs allowed us to narrow down the hypothesis space regarding the source of LIFG dependency effects. In sum, the central aim of this work was to assess whether dependency effects in the LIFG are only elicited for memory-intensive structures involving similarity-based interference or also for “easy” dependencies without much interference. The latter finding would conform to accounts implicating the LIFG for dependency resulting from movement operations (or long-distance dependency itself) whereas the emergence of LIFG effects only in the presence of interference would suggest a more memory-driven role. Note though that the interpretation of “movement” always involves retrieval whether or not the movement configuration places any extra burden on working memory. Thus a uniform effect of “movement” on the LIFG could reflect a generic role in retrieval, as opposed to a (language-) specific one in movement.

### Retrieval Interference in Behavior and the LIFG

Enhanced LIFG activity for ORs as compared with SRs aligns with behavioral effects of increased processing time for ORs over SRs. However, recent behavioral studies have suggested that the retrieval process in ORs may only be more costly than that of SRs under conditions that engender retrieval interference (Gordon et al., 2001, 2002; Fedorenko et al., 2006; Van Dyke and McElree, 2006, 2011; Lee et al., 2007; Hofmeister, 2011). Evidence suggests that sentence comprehension relies upon a cue-driven, direct-access operation (e.g., McElree, 2000; McElree et al., 2003; Martin and McElree, 2008, 2009, 2011), in which cues formed at the retrieval site make contact with representations in memory that have matching content, without the need for a search process. Direct access is performed quickly, but can be highly susceptible to interference (Foraker and McElree, 2011). Basic memory research, as well as research on the role of memory in comprehension, indicates that the primary locus of interference occurs during retrieval (Van Dyke and McElree, 2006). Retrieval interference can result from “cue-overload,” a condition where retrieval cues are not distinctive enough to reliably elicit a desired target because they were associated with multiple items in memory (Watkins and Watkins, 1975; Nairne, 2002; Öztekin and McElree, 2007). In these circumstances, a sought-after element in memory may fail to be recovered, another element matching the retrieval cues may be recovered in its place, or “blend errors” may occur where two or more representations are “synthesized at retrieval” (Nystrom and McClelland, 1992).

It is natural to expect retrieval interference to be a key determinant of whether comprehension is successful. One type of retrieval interference that may impede the processing of OR dependencies is similarity-based interference: i.e., when two adjacent or nearby determiner-noun sequences are parallel in their surface syntax (Lewis, 1996). In fact, it has been shown that if the two NPs prior to the verb contrast in their surface structure, then the behavioral OR over SR effect diminishes (Gordon et al., 2001). This suggests that the processing delay is unrelated to the syntactic configuration of ORs. While a full spectrum of features that engender interference remains to be determined, interference has been recurrently observed when memory representations (i) overlap in their semantic category membership (Gardiner et al., 1972; Wickens, 1973; Dillon and Bittner, 1975; Watkins and Watkins, 1975; Crowder, 1976); (ii) have similar phonological forms (Haber and Haber, 1982; McCutchen et al., 1991; Acheson and Macdonald, 2011) or (iii) encode similar syntactic structures (Lewis, 1996; Gordon et al., 2001, 2002; Fedorenko et al., 2006; Van Dyke and McElree, 2006; Lee et al., 2007; Hofmeister, 2011).

Similarity-based interference effects have also been reported in neuroimaging studies. Increased activation in the LIFG has been associated with competition resolution in non-language-specific tasks (Thompson-Schill et al., 1997; Brandon et al., 2003, 2004; Derrfuss et al., 2004; Postle et al., 2004; Feredoes et al., 2006), and patients with lesions in this region have shown deficits when performing non-syntactic interference tasks (Costello and Warrington, 1989; Robinson et al., 1998; Thothathiri et al., 2010). This suggests that Broca’s area should not merely be linked with syntactic processing, but instead plays a key role in resolving more domain-general interference.

In a previous study, we employed the time course sensitive technique of MEG to link the behavioral finding that OR effects depend on structural parallelism to the LIFG literature. Specifically, we investigated whether the LIFG effect would also be reduced when structural similarity between pre-verbal NPs is removed (Leiken and Pylkkänen, 2014). Our findings indicated that this was indeed the case; LIFG effects of similarity-based interference—but not the pure presence of a dependency—were found at the gap site in ORs. Thus, it was shown that MEG could indeed be employed for the study of object extraction, revealing effects at the gap site around 600 ms after verb onset; a time window consistent with the time course of EEG findings for dependency formation (King and Kutas, 1995; Kaan et al., 2000; Gouvea et al., 2010). Moreover, these results were consistent with working memory and/or conflict resolution-based hypotheses of the role of the LIFG, as opposed to purely syntactic accounts.

### Three Constructions: Object Relatives, Verb Phrase Ellipsis, and Right Node Raising

#### Object Relative Clauses

In the current work, we engaged in a more large-scale investigation of the relationship between dependency formation and similarity-based interference. While the findings from our previous study—an LIFG increase only for ORs containing competing determiner-NPs—are consistent with a similarity-based retrieval interference account of LIFG activity (Öztekin et al., 2008, 2009), they do not yet conclusively rule out movement theories that LIFG activity increases for materials that contain a dependency resulting from a movement operation (Grodzinsky, 1986, 2000; Ben-Shachar et al., 2003, 2004; Grodzinsky and Friederici, 2006; Santi and Grodzinsky, 2007; Grodzinsky and Santi, 2008). Because of the predictable nature of an OR dependency, the lack of a gap-site effect in the LIFG for ORs without determiner-noun competitors may be due to the fact that dependency processing could primarily take place before the verb, prior to the gap. In fact, prior ERP studies have not only revealed P600 effects following the verb, but also a sustained anterior negativity at the point of the filler item in ORs prior to the verb (Phillips et al., 2005). This result could suggest that the bulk of dependency processing may occur in an anticipatory time window, preceding the completion of the gap-filling computation. Thus, one goal of the present study was to investigate whether the predictability of OR dependencies yields early effects in the LIFG, consistent with the dependency effects found in studies of movement theories, or whether ORs truly only elicit LIFG increases as a result of similarity-based interference. To address this question, the present MEG study (i) analyzed LIFG activity in earlier time windows prior to the gap site and (ii) compared LIFG activity elicited by ORs to LIFG activity elicited by non-predictable dependencies. With regards to the analysis of pre-gap LIFG activity, we employed OR clauses inside of sentence structures such as (3) to allow for more natural stimuli than in Leiken and Pylkkänen (2014).

(3) The husband hogged *the blankets that Jane grabbed* after ward.

The relative pronoun *that* may act as a signal that the object *the blankets* will be employed later on in a gap-filling dependency. It is possible that there are initial steps involved in computing a dependency, which may be able to be completed as soon as the filler item is recognized, even prior to the detection of a gap. Thus, once the gap is encountered, a sufficient portion of the processing sequence has been completed such that significant LIFG increases at the gap site would not be found. As this may have been the case in our previous study, in addition to measuring LIFG activity following the target verb *grabbed* prior to the gap site, the present study also analyzed activity in the earlier time window, following *that*, where predictive processing of the upcoming gap may take place.

Notice that the item of retrieval is a determiner-noun *the blankets*, which is different in its surface structure from the nearby proper name *Jane*. In order to investigate whether LIFG activity results from similarity-based interference, we included OR conditions which, as in the previous study, allow for potential similarity-based inference between these two phrases by replacing *Jane* with a second determiner-NP (e.g., *the wife*).

To investigate whether LIFG activity, specifically at the gap site, results from a gap-filling process, non-gap-filling depen dency constructions, such as (4), were employed as controls.

(4) The husband hogged the blankets and Jane grabbed *them* afterward.

Here, as there is no movement, there is no gap-filling dependency. However, it is important to note that the pronoun *them*, which replaces the gap in the OR construction, also forms a dependency with *the blankets*. Therefore, we might expect that a similar retrieval process occurs between retrieval at a pronoun and retrieval at an OR gap, and thus a comparison between condition (3) and condition (4) would yield little difference in LIFG activity. However, as we are using MEG, we will have the time course sensitivity to target activity immediately following the word *grabbed* in both conditions. We expect retrieval to be taking place in ORs during this time window, but later at *them* in controls. Furthermore, the results from Santi and Grodzinsky (2007) suggest that gap-filling shows a cost in the LIFG that retrieval at a pronoun does not. Therefore, we might expect increases for ORs in the LIFG over controls.

#### Verb Phrase Ellipsis

For the comparison of LIFG activity at the gap site in predictable ORs to the gap site of a non-predictable dependency, we employed a gap-filling dependency that does not contain a relative pronoun-like cue to the upcoming dependency; namely, verb phrase ellipsis (VPE). VPE is a two-clause construction that contains an overt verb phrase in the first clause, which, in the second clause, is interpreted, but replaced by an auxiliary verb. For example in (5), the dependency is between the overt verb phrase *called a cab* in the first clause and the gap resulting from ellipsis of the VP in the second clause.

(5) The pedestrian [called a cab]_i_, and the bellhop did *t*_i_ too (Martin and McElree, 2008).

Like the retrieval of the filler at the gap site in ORs, the first-clause VP is retrieved later in the sentence. However, unlike ORs, VPE has no grammatical marking, like a relative pronoun, signaling that the verb phrase in the antecedent has a further role downstream (Martin and McElree, 2008). Without an early indication of a dependency, we expect the bulk of dependency-related neural activity to obligatorily take place after the ellipsis cue has been encountered. The present study, therefore, employs VPE constructions like (6), which will be analyzed following the ellipsis site, at *too*, in comparison with the OR gap site.

(6) The husband *hogged the blankets* and Jane did too.

If LIFG activity in response to gap-filling is reflective of a predictive process, then we expect ORs to show LIFG increases prior to the gap site, whereas we expect VPE to show LIFG increases following the ellipsis site.

On the other hand, LIFG activity for gap-filling constructions has previously been attributed to similarity-based interference. Therefore, to test this hypothesis this study included VPE conditions which contain a competitor for the VP item of retrieval. While a large literature exists for similarity-based interference in ORs, there is little precedent for what might induce this type of interference in VPE. Therefore, in order to introduce a rival VP, the present study interpolated an inner relative clause within VPE constructions, as in (7):

(7) The husband *hogged the blankets* and the wife who sometimes *nagged him* did too.

In this construction, the inner relative clause, *the wife who sometimes nagged him*, involves a VP *nagged him*, which may compete with *hogged the blankets* during retrieval at *too*. For consistency, similarity-based interference conditions of ORs also included these inner relative clauses. It is important to note that this yields parallel OR materials where one of the parallel NPs will contain an inner relative clause, while the other does not. This could potentially lower the similarity between these phrases, thus biasing against possible similarity-based interference effects in these conditions over non-parallel conditions.

According to Martin and McElree (2008), the information inside of the ellipsis site is not necessarily a structurally identical copy of the antecedent VP, as previously suggested (Frazier and Clifton, 2001). Instead, there was evidence that ellipsis may be interpreted using direct-access content cues. In this case, working memory will use a “pointer” mechanism to access the information in the antecedent. Therefore, measurements at *too* should essentially indicate the cost in the LIFG of this “pointing” mechanism. For comparison with a non-ellipsis construction, the present study will include controls, such as (8):

(8) The husband hogged the blankets and Jane did *that* too.

Note that this condition involves the pronoun *that* prior to the retrieval site. This pronoun forms a dependency with the VP from the first clause *hogged the blankets*. This pointing back to the antecedent VP is very similar to that in ellipsis. However, in VPE conditions the pointing is taking place at *too*, whereas in the controls this retrieval has already been completed at *that*. Therefore, a comparison at *too* might show increases for the ellipsis pointer mechanism over the control condition.

#### Right Node Raising

Object relatives and VPE not only differ in terms of their predictability, but also in terms of the syntactic category of their item of retrieval. That is, whereas ORs involve retrieval of an object or individual, VPE involves retrieval of a verb phrase. Therefore, any differences found in LIFG activity between these two constructions may not necessarily be attributable to predictability differences, but may be reflective of the difference in item retrieval. To control for this potential confound, we included a third predictable gap-filling construction, which also involved a dependency between a gap and verbal element; right node raising (RNR). There are several competing accounts of RNR, including those that liken it to ORs^1^ and others that associate it with VPE.^2^ At present, however, it is a rather understudied construction, particularly in terms of how it is processed. Thus, we made a number of assumptions regarding the processing of RNR constructions.

(9) The husband *hogged* and Jane grabbed the pillows.

In (9), the verb in the first clause, *hogged*, requires an object, and the conjunction *and* indicates that what will follow will either be a VP-conjunct for *hogged*, or a larger DP-VP clause parallel to the one already presented. Therefore, when the DP, *Jane*, is encountered, it may lead to the expectation for the upcoming VP-conjunct, *grabbed the pillows*, which is parallel to the verb-gap phrase in the first clause. As a result, the object of the VP-conjunct in the second clause, *the pillows*, may be shared by the VP in the first clause. Under this set of assumptions, the item retrieved at the filler item, *the pillows*, is the verb-gap phrase *hogged*. This type of retrieval links RNR with VPE, which both have verbal items of retrieval, in contrast with ORs. It’s important to note, however, that RNR is like ORs in terms of another property: predictability. If, as described above, the conjunction *and* followed by a DP acts as a cue to the upcoming VP, this would suggest that it is possible to begin processing the upcoming gap-filling computation prior to encountering the filler. Therefore, RNR will be included in the present study as a control for potential confounds, as it equates to VPE in terms of item of retrieval. Because of this shared property, in addition to the lack of existing literature on RNR processing, introducing the potential for similarity-based interference will be done in the same manner as VPE. That is, an inner relative clause will be interpolated, including a VP competitor for the item of retrieval, as in (10):

(10) The husband *hogged* and the wife who sometimes *nagged him* grabbed the pillows.

RNR will be analyzed at the retrieval site, *the pillows*, to examine similarity to VPE. Additionally, LIFG activity in the predictive region, *the wife*, will be analyzed. If RNR constructions are similarly predictable to ORs, then we might expect the bulk of LIFG activity to take place prior the filler item. RNR is also unique in that the gap precedes the filler, a configuration that is novel to the neurolinguistic literature. This reverse ordering of dependent elements might indicate a potential difference in the neural response between gap-filler RNR and filler-gap constructions.

In sum, using the temporal resolution of MEG, our aim was to assess to what extent the LIFG effect of dependency formation is modulated by predictability and/or syntactic similarity, in order to adjudicate between the multiple competing accounts of LIFG involvement in long-distance dependencies. Specifically, if the LIFG does not participate in dependency formation operations *per se*, but rather domain-general operations involved in retrieval and/or competition resolution, then LIFG effects should be modulated by similarity-based interference; specifically, conditions with the potential for high similarity-based interference should show strong LIFG effects. Additionally, if the absence of dependency effects in ORs without high similarity in Leiken and Pylkkänen (2014) was due to pre-gap predictive processing, then we would expect LIFG effects following the relative pronoun cue *that* in ORs. An unpredictable filler-gap construction, like VPE, which does not contain a cue to the upcoming dependency, would not allow for such predictive processing as in ORs. Therefore, we would expect LIFG effects to be delayed in VPEs until the late indication that ellipsis has taken place. Finally, RNRs, which enable prediction—albeit of a filler, rather than a gap—should pattern with ORs in allowing for pre-dependency LIFG effects. On the other hand, any similarity between RNR and VPE constructions (in contrast with ORs) would likely reflect retrieval of a verbal element as opposed to retrieval of an object. Unlike hemodynamic techniques, MEG provides the millisecond-by-millisecond temporal accuracy to attribute effects to specific portions of a trial. Thus, we can with confidence assess whether these effects are predictive of the upcoming gap, or result from encountering the gap.

Finally, it should be noted that although our region of interest (ROI) will simply be referred to as the LIFG, it is nowadays well-known that the LIFG (or “Broca’s Area”) is in fact a grouping of sub-regions with heterogeneous functionality, consisting of at least three Brodmann’s areas (44, 45, and 47) and potentially further subdividing into multiple smaller regions according to evidence from multiple receptor mapping (Amunts et al., 2010). While both areas 44 and 45 have been implicated in sentence processing involving syntactic interference (e.g., Stowe et al., 1999; Cooke et al., 2002; Fiebach et al., 2004; Makuuchi et al., 2009), some linguistic competition tasks have distinguished between the two subparts, affecting only the pars triangularis/BA 45 (Gough et al., 2005; Guo et al., 2010) or only the pars opercularis/BA 44 (Mead et al., 2002; Gough et al., 2005). Crucially, MEG is unlikely to be able to distinguish between areas 44 and 45, and thus these two areas have been collapsed into a single region in our analysis. Therefore, our results will not inform any possible functional subdivision among these regions.

## Materials and Methods

### Participants

Twenty-two right-handed native English speakers participated in the study (13 female; average age: 24.95 years). All had normal or corrected-to-normal vision. The study was formally approved by the New York University Institutional Review Board and all participants gave written informed consent.

### Stimuli and Task

As shown in **Table 1**, three different construction types were investigated: ORs (e.g., *The husband hogged the blankets that Jane grabbed afterward*); VPE (e.g., *The husband hogged the blankets and Jane did too*); and RNR (e.g., *The husband hogged and Jane grabbed the pillows*). Each type has two forms; one involving nearby elements that are parallel in their surface syntax to induce similarity-based interference (“par”), and one that contains elements which differ in their surface syntax (“nonpar”). Parallel types contain an interfering element designed to compete with the element being retrieved at a “gap” site. That is, a parallel NP in ORs, a parallel verb in RNR, and a parallel verb phrase in VPE. Sixty proper names (e.g., *Jane*), one for each set of non-parallel conditions, were employed. These names were replaced by a determiner-NP in parallel conditions (e.g., *the wife*). A non-dependency counterpart of each type was also included. This yielded a 2 × 2 design with similarity and dependency as factors within each construction type.

**Table 1:** Experimental design with the critical items of MEG analysis bolded.

OR	Non-parallel	DEP	The husband hogged the blankets that Jane **grabbed** afterward
		Control	The husband hogged the blankets and Jane **grabbed** them afterward
	Parallel	DEP	The husband hogged the blankets that the wife who sometimes nagged him **grabbed** afterward
		Control	The husband hogged the blankets and the wife who sometimes nagged him **grabbed** them afterward
VPE	Non- parallel	DEP	The husband hogged the blankets and Jane did **too**
		Control	The husband hogged the blankets and Jane did that **too**
	Parallel	DEP	The husband hogged the blankets and the wife who sometimes nagged him did **too**
		Control	The husband hogged the blankets and the wife who sometimes nagged him did that **too**
RNR	Non- parallel	DEP	The husband hogged and Jane grabbed **the pillows**
		Control	The husband hogged the sheets and Jane grabbed **the pillows**
	Parallel	DEP	The husband hogged and the wife who sometimes nagged him grabbed **the pillows**
		Control	The husband hogged the sheets and the wife who sometimes nagged him grabbed **the pillows**

The specific conditions included: (i) sentences containing VPE (ellipsis-par; ellipsis-nonpar), (ii) VPE-controls containing *that* instead of ellipsis to point to the antecedent (ellipsis-control-par, ellipsis-control-nonpar), (iii) sentences containing ORs (OR-par, OR-nonpar), (iv) OR-controls containing a conjunction instead of a complementizer (OR-control-par; OR-control-nonpar), (v) sentences containing RNR (RNR-par; RNR-nonpar), (vi) RNR-controls in which an NP was inserted in the gap site resulting in a basic conjunction (RNR-par; RNR-nonpar), and (vii) filler sentences without syntactic dependencies for variability (filler-par; filler-nonpar). Each condition consisted of 60 trials, so altogether, each participant viewed 840 trials. The targets of MEG analysis were the dependency formation sites themselves (gap site in ORs, ellipsis-site in VPE, and filler-site in RNR) and anticipatory regions.

Obligatorily transitive verbs were used in the first clause of all conditions to prevent interpretation of RNRs as a basic conjunction. “*Did*” (which has an auxiliary verb use) was not used in the second conjunct of ORs/RNR to prevent a VPE reading. The item of retrieval in all conditions (the “filler” in the filler-gap construction), was designed to always been an inanimate object. For example, *the blankets, the bikes*, etc. (A full list of materials containing animate objects can be found in the Appendix.) Some psycholinguistic work has suggested that inanimacy of an object of retrieval may reduce the object-over-SR clause effect (Traxler et al., 2005), as well as neuroimaging work (Chen et al., 2006). Therefore, because the filler item was inanimate across all our sentence types, any effect of this type should be equivalent across conditions. Further, it should be more difficult to find an effect of similarity-based interference for ORs in the event that inanimacy diminishes processing load of OR clauses.

To ensure that the complexity of our materials did not sacrifice plausibility, we collected plausibility judgments on our stimuli using the Amazon Mechanical Turk (AMT) interface prior to the MEG recording (see Appendix 1). The test stimuli described above were complemented with syntactically grammatical, but highly implausible stimuli for comparison, following designs which similarly compare grammatical, but complex, stimuli with implausible items, such as Pylkkänen et al. (2004). The implausible stimuli were constructed by switching the verb in the first clause in the test items with the verb in the inner relative clause on one-third of the sentences in each condition, resulting in expressions such as *the husband nagged the blankets and the wife who seldom hogged him did too.* We gathered demographic information from 150 participants. Participants were obligated to indicate whether or not they were a native speaker of English and were informed that they were only permitted to participate in the experiment one time. Any participants who violated either of these criteria were rejected from analysis as were any participants who did not fill in the demographic survey. Also, those who far exceeded or fell below the average amount of time taken to complete their list were rejected if extreme durations were accompanied by unreasonable data (e.g., the same response for every trial). Items were distributed among 10 randomized different lists, so each list was completed by 15 subjects. Turk users saw each item and selected a plausibility rating on a 0–7 Likert scale (0 = completely implausible). Participants’ raw ratings were averaged over each condition.

A *t*-test comparing the stimuli to be included in the MEG recording with implausible fillers showed that experimental stimuli (*M* = 5.1408) were rated significantly higher (*p* < 0.001) than their implausible counterparts (*M* = 1.9199) on a 0–7 scale, suggesting that the sentences intended for the MEG study were considered generally plausible.

Unsurprisingly, our stimulus manipulation affected the plausibility ratings, with a 2 × 2 × 3 ANOVA on the critical stimuli showing reliable main effects of all three factors. These effects were driven by lower ratings for parallel than non-parallel stimuli [Parallelism: *F*(1,708) = 176.26, *p* < 0.001; non-parallel *M* = 5.51, parallel *M* = 4.81], for dependency than control stimuli [Dependency *F*(1,708) = 179.59, *p* < 0.001, dependency *M* = 4.80, control *M* = 5.51], and by higher ratings for VPE than the other two constructions [Construction type: *F*(2,708) = 41.858, *p* < 0.001, VPE *M* = 5.39, OR *M* = 5.25, RNR *M* = 4.83].

The main effect of Parallelism was significant within each construction (all *F*s > 25) though it was most robust within the OR-dependencies, as reflected by a reliable 2 × 2 interaction between Parallelism and Dependency within the ORs [*F*(1,236) = 18.413, *p* < 0.001, non-parallel control *M* = 5.95, parallel control *M* = 5.29, non-parallel dependency *M* = 5.57, parallel dependency *M* = 4.21], while no such interaction was observed within VPE [*F*(1,236) = 1.998, *p* = 0.159, non-parallel control *M* = 5.45, parallel control *M* = 5.03, non-parallel dependency *M* = 5.87, parallel dependency *M* = 5.23] or RNR [*F*(1,236) = 0.072, *p* < 0.788, non-parallel control *M* = 5.97, parallel control *M* = 5.38, non-parallel dependency *M* = 4.25, parallel dependency *M* = 3.72]. The three way interaction between Parallelism, Dependency and Construction was also significant [*F*(2,708) = 4.43, *p* = 0.01].

The main effect of Dependency was qualified by an interaction with Construction [*F*(2,708) = 120.19, *p* < 0.001], with reliably decreased ratings for dependency than control sentences for OR [*F*(1,236) = 79.86, *p* < 0.001; control *M* = 5.62, dependency *M* = 4.89] and RNR [*F*(1,236) = 238.74; *p* < 0.001, control *M* = 5.67, dependency *M* = 3.98], while the reverse held for VPE [*F*(1,236) = 14.902, *p* < 0.001; control *M* = 5.24, dependency *M* = 5.55].

In sum, parallelism uniformly decreased plausibility ratings, while the presence of a dependency decreased judgments for ORs and RNR but not for VPE. Thus any LIFG patterns tracking these effects could reflect plausibility instead of the independent variables of interest; we return to this in our report of the results.

During the MEG recordings, participants read all critical stimuli that were included in the MTurk study (with the exception of the implausible stimuli). Presentation was word-by-word (except in the case of determiner-NPs which were presented as a unit for time restriction purposes, e.g., *the wife*). After one-third of the linguistic stimuli, participants were presented with a comprehension question relating to the content of the previous text (e.g., *Did the husband grab the pillows?*) to which the answer was either “yes” or “no.” For the purposes of this task, the participants were given practice outside the MEG machine and again inside the MEG machine prior to recording. Half of the questions had the answer “yes” and half had the answer “no.” For a “yes,” both the character and the action mentioned in the question needed to match those in the previous text.

### Procedure

**FIGURE 1:**
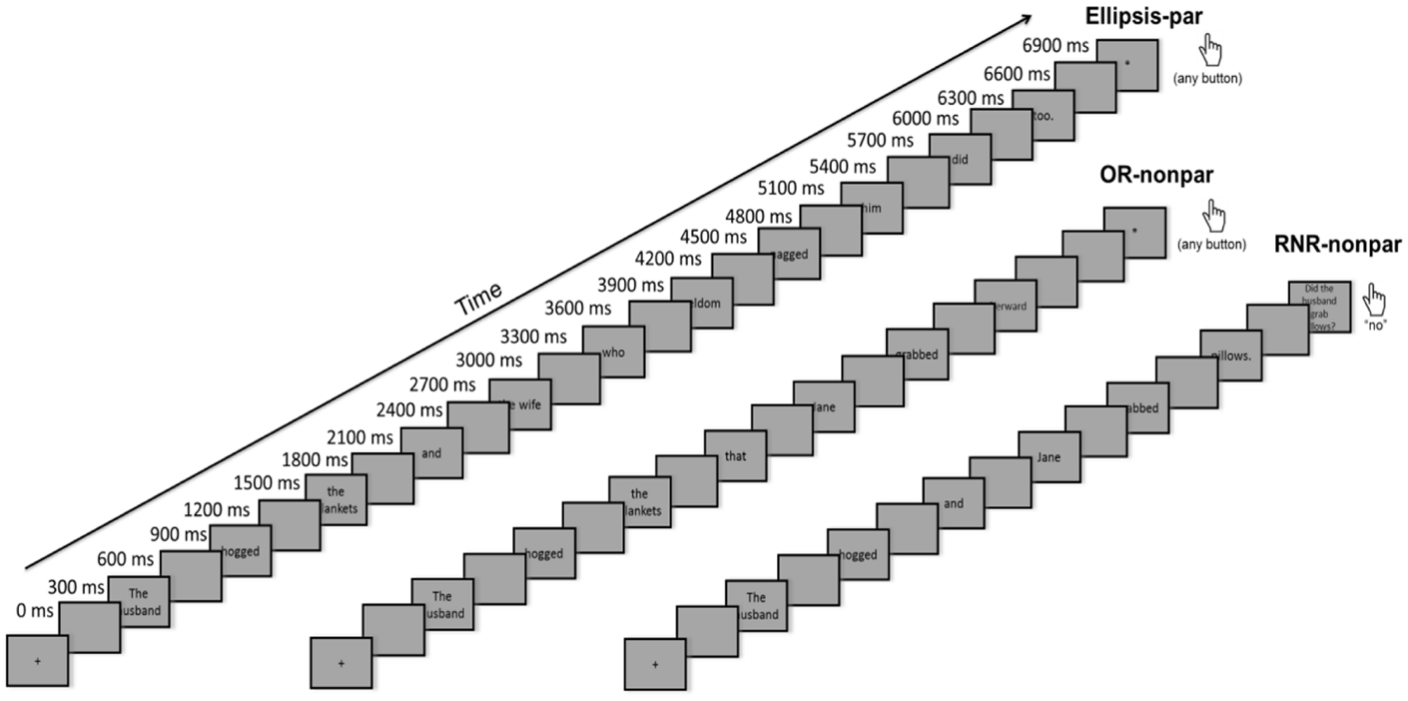
**Trial structure**.

Before the MEG recordings, participants were instructed about the experimental task and their head shapes were digitized using a Polhemus (Colchester, VT, USA) FastSCAN COBRA 3D laser system. During the experiment, participants lay in a dimly lit, magnetically shielded room (Vacuumschmelze, Hanau, Germany). Using PsychToolbox, the experiment was presented on a 7x7-inch screen with a resolution of 1024 × 768 pixels placed approximately 9.5 inches above the subjects’ eyes. Stimuli were presented word by word, 300 ms for each word, with a 300 ms blank screen between each word. To allow for longer processing time of complex stimuli, a blank screen was then presented for 700 ms prior to the question screen. Using a button press, the subject expressed whether the answer to the comprehension question was “yes” or “no” (**Figure 1**). Trial order was random. Subjects were in the machine for an hour, with five breaks (between each of the six blocks), and were then given an extended break outside of the MEG room, due to the length of the study. Subjects then returned to the machine for the next six blocks. The entire recording took about 2.5 h. MEG data were collected using a using a whole-head 157-channel axial gradiometer system (Kanazawa Institute of Technology, Nonoichi, Japan). For this study, data were recorded at a sampling rate of 1000 Hz with a low-pass filter at 200 Hz using a DC recording and a notch filter at 60 Hz. Eye-blinks were recorded using an SR Research Eyelink 1000 Arm-Mounted Eyetracker sampling at 1000 Hz.

### Data Analysis

#### Pre-processing of MEG Data

Raw data were noise-reduced (CALM; Adachi et al., 2001) and cleaned of artifacts (at a threshold of 4000fT). On average, no more than 25% of trials were lost during this procedure. Artifacts also included eye-blinks, which were removed by aligning the eye-tracking recording (described above) with the MEG recording. Data were high-pass filtered at 1 Hz. Data were then averaged by condition using a 200 ms pre-stimulus interval and a 1000 ms post-stimulus interval and baseline corrected using the 200 ms pre-stimulus interval. Data were low-pass filtered at 40 Hz after averaging, using the program BESA^®^ 5.1 (MEGIS Software GmbH). Additionally, one subject was excluded as an outlier due to excessive blinking.

#### ROI Analysis of Minimum Norm Estimates

Magnetoencephalography data were analyzed as distributed sources using L2 minimum norm estimates calculated in BESA. The minimum norm images were depth weighted as well as spatiotemporally weighted, using a signal subspace correlation measure (Mosher and Leahy, 1998). LIFG activity at the site of dependency formation (OR: *grabbed*; VPE: *too*; RNR: *grabbed*) was examined via an ROI analysis. For the ROI analysis, sources were assigned to the anatomical LIFG region consisting of Brodmann’s areas left 44 and 45, based on coordinates in Talairach space (Lancaster et al., 2000). Non-parametric, cluster-based permutation tests (Maris and Oostenveld, 2007) were performed in the same time windows as in Leiken and Pylkkänen (2014); an early “N400”-like time window (200–500 ms), associated with lexical access (Embick et al., 2001; Pylkkänen et al., 2002; Pylkkänen and Marantz, 2003), and basic combinatory effects (Bemis and Pylkkänen, 2011), and a late “P600” time window (500–800 ms) associated with OR versus SR P600 effects (Kaan et al., 2000). Additionally, due to the length and complexity of the current study’s stimuli, a third, even later, analysis window was added (800–1000 ms). Permutation tests were employed to identify temporal clusters significantly affected by stimulus manipulation, corrected for multiple comparisons. Thresholds for initial cluster selection followed Leiken and Pylkkänen (2014), i.e., of waveform separations that lasted for 10 or more time points at *p* < 0.3, the one with the largest summed *F* or *t* statistic within each time-window was entered into 10,0000 permutations. The final corrected *p*-value for each cluster was calculated as the ratio of permutations yielding a test statistic greater than the actual observed test statistic (α = 0.05). The tests were a 2 × 2 repeated measures ANOVA (Similarity × Dependency) over each time window (“N400,” “P600,” and late response) within each construction type at the site of dependency formation: at the onset of the verb preceding the gap in ORs, at the onset of the verb preceding the filler in RNR, and at the onset of the word following the ellipsis in VPE. The epochs were set to begin from the onset of each of these words through the following word. This extension allowed us to detect potential residual dependency effects which may have occurred early in the processing of the subsequent word. The ANOVA was then followed up with planned pairwise comparisons between parallel versus non-parallel subtypes, and dependency versus control subtypes. Effects at *p <* 0.05 will be discussed as significant and effects between this corrected level and *p* < 0.10 as marginal. Any *p*-values higher than this will be considered numerical trends. Our conclusions will, however, only rest on results reaching corrected significance at *p* < 0.05.

The above tests were followed by analyses at the pre-dependency time intervals (i.e., at the filler in ORs following the relative pronoun cue *that*, after the gap in RNR, and at a comparable lexical item in VPE). That is, analyses were performed in windows where the potential effects of dependency prediction may have taken place (i.e., prior to the gap in ORs, prior to the filler in RNR, and prior to the auxiliary verb in VPE). Unlike at the OR gap site, the lexical material in the predictive region was not matched in all four OR conditions (*that* in parallel and *and* non-parallel) Therefore, parallel and non-parallel ORs, along with their control counterparts could not be included in a single 2 × 2 ANOVA as above. Preverbal material was instead submitted to *t*-tests in order to examine potential anticipatory dependency processing in the same LIFG region. *t*-tests were performed after the presentation of the filler item: e.g., *the wife* in parallel conditions and *Jane* in non-parallels in the examples in **Table 1**. If effects of dependency prediction only occur in conjunction with similarity-based interference, then a difference would only be found between the two instances of *the wife* and not between the two instances of *Jane*. However, if anticipatory LIFG effects are independent of parallel syntactic structure, then both *t*-tests should show a difference. In RNR, the immediate post-gap lexical item *and* is the same in all four conditions, allowing for a 2 × 2 (similarity × dependency) ANOVA. This was then followed up by *t*-tests on the next word comparable to those performed on ORs; at *the wife* in parallel conditions, and *Jane* in non-parallel conditions. In VPE, no dependency distinction exists between the four conditions until after auxiliary verb. To confirm that no LIFG effect of dependency anticipation occurs in a time window prior to *did*, a 2 × 2 ANOVA at the conjunction *and* was performed. As in the ORs and RNR, *t*-tests were also performed within parallel conditions at *the wife* and within non-parallel conditions at *Jane*, to confirm the assumption that having no cue to an upcoming dependency prohibits dependency prediction. The *t*-tests employed the same settings as the above 2 × 2 ANOVAs; 10,000 permutations with the same cluster thresholds within the same three time intervals.

#### Full Brain Analyses

The ROI analyses were each supplemented by liberally thresholded uncorrected full brain contrasts. The goal of these analyses was to confirm that the effects found in the ROI analyses in fact reflected activity localized with the LIFG (as opposed to spillover from neighboring regions) and to reveal any other major cotemporaneous effects. We compared the minimum norm estimates of the activity elicited by the experimental conditions sample-by-sample in the same pairwise comparisons described for the ROI analyses. Effects were visualized on the smooth BESA cortex when they remained reliable (*p* < 0.05, uncorrected) for at least three temporal samples and were observed in at least three spatially contiguous cortical sources.

## Results

### Behavioral Data

After one-third of the sentences, participants were asked a comprehension question relating to the content of the stimulus sentence (e.g., Did the husband grab the pillows?) to which the answer was either “yes” or “no.” Subjects performed fairly well on this complex task overall (M = 77.37%), and generally better (average accuracy ± SD) on the non-parallel (M = 82.75 ± 9.78%) than the parallel (M = 72.00 ± 8.12%) trials. In general, performance was slightly higher on control conditions (M = 77.60± 8.94%) compared with dependency conditions (M = 75.60± 9.28%). Performance was quite similar for the dependency version of each construction type: ORs (M = 76.89± 9.04%), VPE (M = 76.99± 9.20%), and RNR (M = 72.92± 9.61%).

### MEG Data

#### Object Relative Clauses

As described above, only one of the two parallel NPs in parallel OR conditions contained an inner relative clause, potentially lessening similarity-based interference in these conditions. Nevertheless, our OR results showed a straightforward though late effect of parallelism after the gap-site, as well as a more complicated effect of dependency, as detailed below. No interactions between our two factors were observed. Test results are considered significant at *p* < 0.05, but for completeness in addressing our hypotheses we will report marginal results and numerical trends resulting from planned comparisons as well. Only significant findings will, however, contribute to our interpretation of results.

The early time window (200–500 ms) showed weak trends both toward a main effect of parallelism and for a main effect of dependency, but neither cluster survived the permutation correction for multiple comparisons (parallelism: *p* = 0.1624 at 397–500 ms; dependency: *p* = 0.7253 at 329–351 ms). The wave form separation during these non-significant main effects did, however, conform to the results of Leiken and Pylkkänen (2014), with only parallel dependencies eliciting increased amplitudes as compared to all other conditions.

**FIGURE 2:**
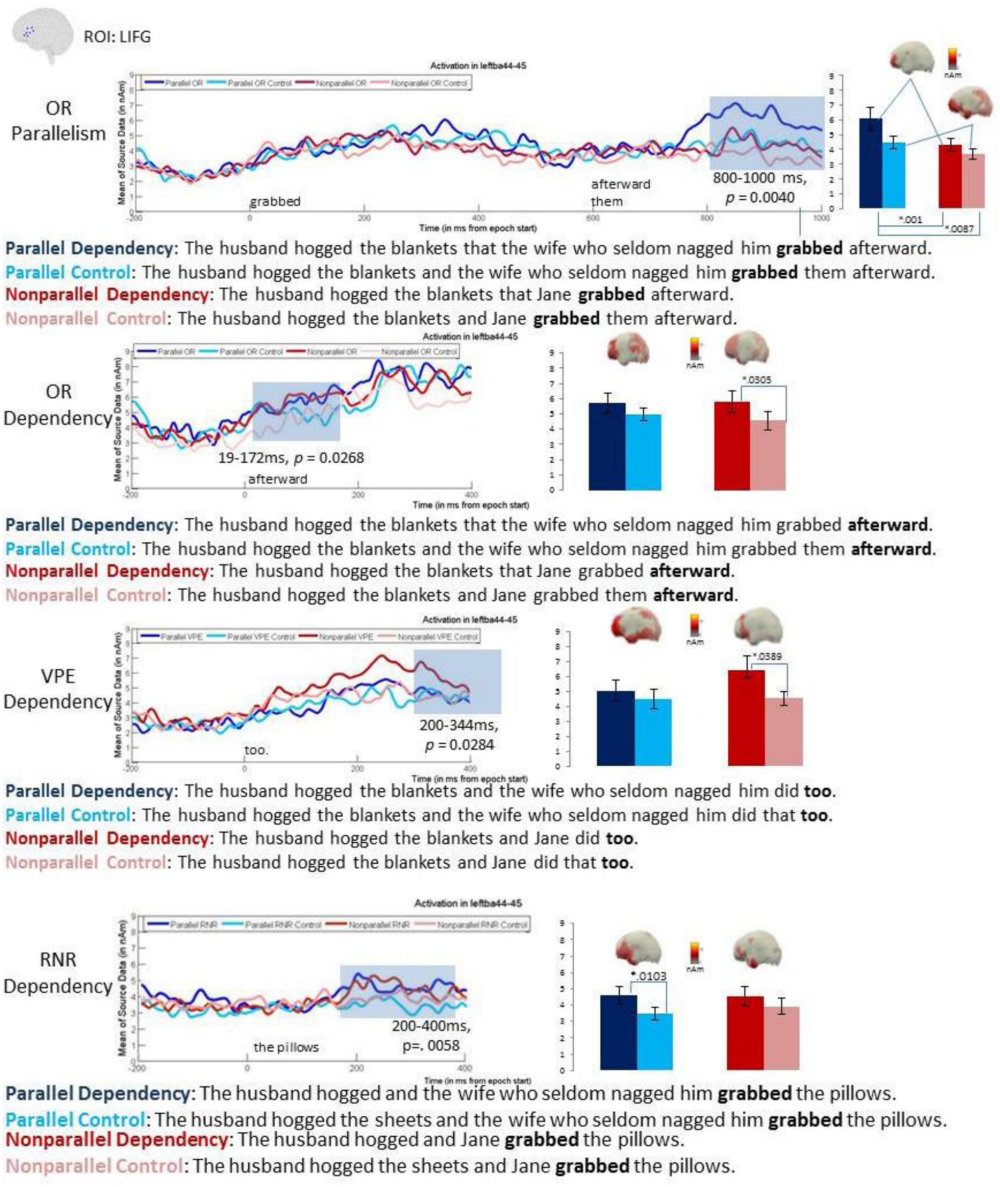
**Left inferior frontal gyrus (LIFG) ROI and Whole Brain results.** The clusters of time points that were reliable in a cluster-based permutation *t*-test are shaded gray showing the LIFG increase for the dependency conditions as compared with control conditions. Red colors indicate non-parallel conditions, and blue colors indicate parallel conditions. Darker shades of each indicate dependency conditions, whereas the lighter shades stand for controls. Within 19–172 ms in the OR condition, there was a significant increase in the LIFG for OR dependencies versus control conditions (*p* = 0.0268). For the VPE condition, within 200–344 ms, there was a significant LIFG increase for VPE dependencies versus control conditions (*p* = 0.0284). The time window of 200–400 ms shows a significant LIFG increase for RNR (*p* = 0.0058). For each of the three constructions, an accompanying bar graph indicates the means for each condition (parallel dependency, non-parallel dependency, parallel control, non-parallel control) within the time window showing significant dependency increases in the LIFG. ROI findings were well-supported by full brain analyses, confirming LIFG increases within the time window showing significant clusters for each pairwise comparison.

No reliable effects were found in the 500–800 ms time window but the latest time-window, 800–1000 ms, showed both a reliable main effect of parallelism, with the cluster extending throughout this interval (*p* = 0.0041), as well as a reliable main effect of dependency, similarly covering the entire 800–1000 ms interval (*p* = 0.0261; **Figure 2**). These results reflected a pattern of parallel conditions eliciting increased LIFG amplitudes as compared to non-parallel conditions and dependency conditions eliciting increased amplitudes as compared to non-dependency controls. Planned pair-wise comparisons showed that within the dependency conditions, parallel ORs (*M* = 6.16) elicited significantly higher LIFG activity than non-parallel ORs (*M* = 4.373; 800–1000 ms, *p* = 0.0087) whereas within the control conditions, the increase for parallelism (*M* = 4.504) was only marginal (896–970 ms, *p* = 0.0638) versus non-parallel ORs (*M* = 3.643). The effect of dependency trended in the right direction for the parallel conditions (800–863 ms, *p* = 0.1102) but was significant for the non-parallel conditions (895–966 ms, *p* = 0.0366). Parallel ORs also elicited significantly higher LIFG activity than the non-parallel controls (800–1000 ms, *p* = 0.001). In sum, the pairwise comparisons showed increased activity for both dependency conditions over their controls and for both parallel conditions over their non-parallel versions.

However, before we can conclude that the LIFG ROI activity is modulated by the presence of a dependency, a complication arising from the lateness of this effect must be addressed. Namely, the effect occurred during the processing of the word immediately following the target verb; this word being *afterward* in the dependency conditions and *them* in the controls. Thus the LIFG increase could simply have reflected the increased activity for the longer and morphologically more complex *afterward* than *them*. However, since the word after *them* in the control conditions was *afterward* [the full contrast being *grabbed afterward* (OR) versus *grabbed them afterward* (control)], this lexically based explanation would predict that a comparison at *afterward* in the OR versus control conditions should not show the LIFG effect. In contrast, if the LIFG increase at *afterward* in the OR condition reflects dependency processing, it should replicate in a comparison of the two instances of *afterward*. To test this, the baseline was moved to 200 ms before the onset of *afterward* for all four conditions and 2 × 2 permutation ANOVAs were run in the 200–500 ms interval (covering the timing of the effect in the prior analysis) as well as in the 0–200 ms interval, covering any effects occurring at the very onset of this spill-over word. Though the ANOVA for 200–500 ms revealed a cluster replicating the pattern in the prior analysis (i.e., higher amplitudes for dependency than for control conditions), it did not survive correction for multiple comparison. However, in the earlier interval, 0–200 ms, a reliable main effect of dependency was observed (19–172 ms, *p* = 0.0268), with pairwise comparisons showing a significant increase for dependency (*M* = 5.81) over control (*M* = 4.567) within the non-parallel conditions (19–108 ms, *p* = 0.0305) and a similar trend for dependency (*M* = 4.5.692) over control (*M* = 4.4.974) within the parallel conditions (132–164 ms, *p =* 0.1638). These results converge on the finding that the presence of an OR dependency elicited a late LIFG increase occurring after 600 ms post-target verb onset. Interestingly, this effect was stronger for the non-parallel conditions, suggesting that it is not dependent on the presence of parallelism. This is in contrast to the findings of Leiken and Pylkkänen (2014), who for sentence fragments only found an LIFG increase for OR dependencies involving parallel NPs. Thus it is possible that the current full sentential stimuli may have been better test items for detecting a dependency effect in the absence of parallelism.

Given that we did observe an effect of dependency after the gap-site, the prediction for such an effect in the predictive pre-gap region is weakened. In fact no such effects were observed when activity elicited by the filler items (*the wife* in parallels, and *Jane* in non-parallels) was compared in permutation *t*-tests. Thus our findings revealed no evidence for predictive gap-processing in the LIFG.

The whole brain graphs plot the same pair-wise comparisons as reported above on liberally thresholded whole brain minimum norms (time and space thresholds at 3 and *p-*value threshold at 0.05) at the time windows of the significant effects in the ROI analysis. The aim of this analysis was to ascertain that the ROI results in fact correspond to activity localized in the LIFG. These contrasts revealed activity overlapping with the BA 44–45 region during the time window of the parallelism main effect in ORs. Specifically, both parallel ORs and parallel control conditions showed an increase in this region compared with their non-parallel counterparts in the 800–1000 ms time window. The dependency effect early on after the presentation of *afterward* (19–172 ms after the onset) was also observable in the whole brain analyses for parallel ORs over parallel control conditions as well as for non-parallel ORs over non-parallel controls. In addition to left inferior frontal activity, posterior parieto-occipital activation was observed for the parallel control condition over non-parallel controls, as well as in both dependency contrasts.

#### Verb Phrase Ellipsis

For VPE, the cluster-based 2 × 2 ANOVA in the early time-window (200–500 ms) revealed a significant main effect of dependency at 200–344 ms (*p* = 0.0284). As with ORs, this effect was more strongly driven by a pair-wise effect in the non-parallel conditions (280–344 ms, *p* = 0.0389), with dependency (*M* = 5.893) over controls (*M* = 4.330). Parallel conditions showed a weaker trend (*p* = 0.2123 at 259–284 ms) of dependency (*M* = 4.719) over controls (*M* = 4.178). No reliable effect of parallelism was observed in this time-window nor any effects of either factor in the later time-windows. Finally, no effects were found in the pre-gap “predictive” time-windows (at *the wife* within parallels and at *Jane* within non-parallels), consistent with the fact that VPE dependencies are unpredictable.

Full brain analyses were also performed for the pairwise comparisons within the VPE conditions, specifically, contrasts between parallel VPE over parallel controls, and non-parallel VPE over non-parallel controls. These results confirmed to the ROI analyses in revealing LIFG effects within the time window of significant ROI findings. Namely, an effect was obtained at 200–344 ms for parallel VPEs over parallel controls, as well as for non-parallel VPEs over non-parallel controls. Again, the frontal effects were accompanied by more posterior activation in the parallel VPE condition over the parallel control condition, but not in the non-parallel contrast.

#### Right Node Raising

In the RNR analysis, the onset of the second verb (*grabbed* in **Table 1**) was treated as 0 ms, for consistency with the OR analyses. A reliable main effect of dependency was observed in the 800–1000 ms time window (or 200–400 ms following the RNR filler item, *the pillows*; *p* = 0.0058), with the cluster covering the entire analysis interval. In the pairwise comparison this effect was reliable within the parallels (267–400 ms, *p* = 0.0103), with dependency (*M* = 4.600) over controls (*M* = 3.466), and within the non-parallels, only trending in the same direction (299–332 ms, *p* = 0.2251) for dependency (*M* = 4.528) over controls (*M* = 3.920). No other effects were observed, including in the pre-gap “predictive” regions. Thus, like in VPE, parallelism did not appear to affect RNR processing in the LIFG. Timing wise, the RNR dependency effect occurred within 300 ms of encountering the site at which the dependency needs to be formed (which in RNR is the filler). This is similar to the dependency effect in VPE, suggesting that filler-gap order is not a strong modulator of LIFG dependency effects. This timing of course contrasts to the dependency effects observed for ORs, which were much later.

Whole brain pairwise comparisons were performed for the contrasts between parallel RNR and parallel controls, as well as between non-parallel RNR and non-parallel controls, with results conforming to the RNR ROI findings described above. That is, the time window of 200–400 ms after *the pillows*, which showed significant clusters of LIFG activity for parallel RNRs over parallel controls, also reveals significant effects in the full brain contrasts. Similarly, the increase of LIFG activity in non-parallel RNRs over non-parallel controls within 200–400 ms was evident from the full brain plots. For these contrasts, the effects were mostly anterior, though for both contrasts, the LIFG effect appears to be accompanied by an increase in activation in left anterior temporal cortex.

#### Results Summary

In sum, our results indicated an LIFG effect of Dependency for each construction type without interaction with Parallelism, suggesting that this effect is not dependent on similarity-based interference. In ORs, Parallelism had its own main effect, indicating that this factor can drive the LIFG even in the absence of a filler and gap. Importantly, neither effect tracked plausibility as rated in our MTurk norming study: parallelism lowered plausibility judgments across all constructions, but an MEG effect of Parallelism was only obtained for ORs; for Dependency, judgments were lower for dependency conditions in ORs and RNR but higher in VPE while in contrast, LIFG amplitudes increased for dependency conditions regardless of construction.

## Discussion

This study investigated LIFG activity during dependency processing using both a technique and constructions novel to the literature in order to shed light on the role of the LIFG in dependency formation. Our key question was whether dependency effects in the LIFG, whether the result of movement or not, require explicit taxing of working memory via similarity-based interference, or whether the sheer presence of a dependency is sufficient to drive this activity, as predicted by movement-based accounts of activity in this region. Our results show that similarity-based interference is a not a prerequisite for LIFG effects: LIFG amplitudes showed a statistically significant increase when a dependency was present across our three constructions whether or not interference-inducing syntactic parallelism was built into the stimuli. Although sub-types of each construction contributed to the main dependency effect differently, we take the significant main effect within each construction type to show that for these materials, an activity increase was observed in the LIFG for any instance of retrieving the first member of a dependency chain. Thus, contrary to our own previous work, where we used sentence fragments as opposed to full sentences (Leiken and Pylkkänen, 2014), the current results support a role for the LIFG in dependency formation that generalizes across a variety of memory demands. The findings are compatible with the hypothesis that the LIFG computes syntactic movement, but also with the hypothesis that this region has a basic role in retrieval in a variety of non-movement contexts.

Given that Leiken and Pylkkänen (2014) found no purely dependency driven LIFG effects at an OR gap, an important goal for the current study was to investigate whether such effects could be observed in pre-gap LIFG activity, as potential reflections of gap anticipation. However, since here we did find an effect of dependency after OR gaps, the prediction for pre-gap dependency effects was somewhat weakened, and in fact, no anticipatory LIFG effects were observed for ORs or for either of the other construction types. Further, since significant LIFG increases for dependency were observed for ORs, specifically for the non-parallels, even though non-parallel control conditions also contained a type of retrieval at the pronoun *them*, these results conform to prior findings indicating that gap-filling may produce a greater LIFG cost than retrieval at a pronoun (Santi and Grodzinsky, 2007).

Left inferior frontal gyrus effects in OR dependencies were compared with VPE, which contains a non-predictable dependency and was thus anticipated to require the presentation of both filler and gap before the dependency could be processed. The VPE control condition, like that of ORs, also contained a type of non-gap-filling dependency between the pronoun *that* and the VP item of retrieval. Again, MEG time course sensitivity allows for fine-grained measurements at the post-gap word *too* in both conditions, where it was expected that retrieval takes place in VPE, but has already been completed in the control condition at *that*. Both of these expectations were supported, as VPE showed no anticipatory LIFG effects, but did show significant LIFG increases at the ellipsis-site.

While both OR and VPE constructions showed retrieval effects at the gap site in the LIFG, the timing of the effect was much earlier for VPE than that for ORs. While the OR results are compatible with the full time-course of gap-filling processes in previous SAT studies (McElree, 2001; McElree et al., 2003, 2006), their lateness with respect to VPE deserves some attention. Whereas the OR constructions involve retrieval of an object/individual, VPE involves retrieval of a verbal element. Thus, one possibility may be that the category of the retrieved item matters for retrieval time. Another possibility is that operations performed at the retrieval site differ for ORs and VPEs. Our RNR constructions bear on this issue, as they are predictable like ORs, but involve retrieval of a verbal element like VPE. A unique property of RNR constructions, however, is that they contain a gap-filling dependency where the gap precedes the filler, unlike in ORs or VPE. Despite this special property, RNR findings were closely linked with our VPE results. Specifically, RNR showed a significant increase for dependency in the LIFG. Therefore, we cannot attribute VPE-OR differences to predictability, as RNR is matched with ORs for this feature. Interestingly, we note that the similar processing profiles for VPEs and RNRs aligns with the theoretical proposal that RNRs are in fact a type of ellipsis (Wexler, 1980; Swingle, 1993; Kayne, 1994; Wilder, 1997; Hartmann, 2001, 2003; Abels, 2003; Ha, 2008). The slower timing effect for ORs could indicate that the word category of the item of retrieval affects retrieval speed, with access to verbal elements being faster than objects/individuals. Alternatively, and perhaps more plausibly, VPEs and RNRs could be processed more quickly than ORs because, as Martin and McElree (2008, 2009) argued, VPE can be resolved through a pointer mechanism, wherein retrieval consists of pointing to a structure in memory. On the other hand, processing ORs requires building the argument structure of the verb phrase after argument has been retrieved.

Taken together, the present set of findings can, in fact, be accounted for by hypotheses associating the LIFG with dependencies resulting from syntactic movement (Grodzinsky, 2000; Ben-Shachar et al., 2003; Grodzinsky and Santi, 2008), though the main effect of parallelism obtained for the ORs also provides evidence for the role of working memory independent of structure (Caplan et al., 2000, 2008; Fiebach et al., 2001, 2005; Kaan and Swaab, 2002; Rogalsky et al., 2008; Makuuchi et al., 2009) or cognitive control (Botvinick et al., 2001; Miller and Cohen, 2001; Novick et al., 2005; Braver et al., 2007). These results for parallelism are more convincing given that the item of retrieval in all conditions was an inanimate object. In other words, despite the fact that inanimacy has been associated with a reduction in OR processing load (Traxler et al., 2005), similarity-based interference effects were still found for parallel ORs versus non-parallels. Importantly, however, not only did these similarity-based interference effects appear rather late in the present study, at 800–1000 ms as opposed to at 300–400 ms in Leiken and Pylkkänen (2014), but they also only held for ORs, and not VPE or RNR. Regarding latency, it has been shown that the timing of P600 effects can be delayed when dependency length is increased (Phillips et al., 2005). Due to the full sentential stimuli of the present study, the distance between the gap and filler items was much greater than that in our previous study, where we employed minimal OR phrases. Therefore, it is possible that the later timing of similarity-based interference effects was due to the large amount of mediating material between filler and gap. The high complexity of the present study’s materials may also be relevant for the fact that the parallelism effects were only found for ORs. That is, the ORs were contained inside of a complex sentential structure, as in (3), where the first constituent of the sentence, the matrix subject, (e.g., *the husband*) was of the same determiner-noun structure as the item of retrieval (e.g., *the blankets*) and its competitor (e.g., *the wife*).

(1) *The husband* hogged *the blankets* that *the wife who sometimes nagged him* grabbed afterward.

This property may have induced *proactive* interference with the item of retrieval, where material prior to the initial encoding of the target item creates competition with it (Öztekin and McElree, 2007). The VPE and RNR stimuli did not have elements inducing possible proactive interference. This factor may also have contributed to the latency of the OR retrieval effect as compared with the other two conditions which showed similar effects in a much earlier time window.

In sum, our two main results are LIFG increases in response to similarity-based interference in ORs, and LIFG increases in response to the presence of the three different dependency types regardless of similarity-based interference. While both of these findings are attributed to “LIFG” activity, it is important to note that this region contains heterogeneous subparts. Therefore, it is possible that the interference effect is in one subdivision, whereas the effects for retrieval are in the other. The spatial resolution of MEG is, however, unlikely to be able to disambiguate the detailed localization of these effects within the LIFG and thus we must leave this question for future work.

## Conclusion

This study took advantage of the detailed time-resolution of MEG and the stimulus properties of three different dependency constructions–ORs, VPE, and right-node-raising—to target several of the major competing accounts of the role of the LIFG in dependency processing. Our findings revealed that at the retrieval sites of each of these three dependencies, LIFG increases are observed, conforming to “movement” accounts. Additionally, in ORs only, effects of similarity-based interference were observed in the LIFG, consistent with working memory or cognitive control theories. Thus, our results add to the growing body of evidence that a complete understanding of “Broca’s Area” must take into consideration both structure and memory related processes. Overall, our results are consistent with a hypothesis that the LIFG region subserves the recovery of an element from memory. The exact generality of this process across contexts remains a question for future work, but the current results enable a new level of temporal and computational precision in subsequent hypotheses about the type of retrieval that the LIFG contributes to.

## Conflict of Interest Statement

The authors declare that the research was conducted in the absence of any commercial or financial relationships that could be construed as a potential conflict of interest.

## Footnotes

1 The gap-filling dependency of RNR is considered similar to ORs, in that it may involve the extraction of an element, resulting in a link between its overt position and its interpretation in the original location. While both ORs and RNR might involve extraction to a higher location, the extracted item in an OR undergoes leftward movement, whereas the extracted element in RNR would be moving rightward (Ross, 1968; Bresnan, 1974; Postal, 1974, 1998; Sabbagh, 2007). Therefore, the gap left behind in ORs follows the extracted item, whereas the gap left behind in RNR *precedes* the extracted item, as in the below example: Some people love [t]_i_, but other people hate [t]_i_, [the role that government plays in this country]_i_. (Postal, 1974).

2 On the other hand, non-movement analyses of RNR suggest that the shared object is simply an overt argument of the second clause, but deleted due to identity in the first clause. This representation of RNR would be more analogous to ellipsis accounts, which, as explained above, delete the VP in the second clause due to redundancy.
