# Supplementary material for: Filling Predictable and Unpredictable Gaps, with and without Similarity-Based Interference: Evidence for LIFG Effects of Dependency Processing
Source: Front Psychol. 2015 Nov 16;6:1739. doi: 10.3389/fpsyg.2015.01739 (PMC4644799; doi:10.3389/fpsyg.2015.01739)
Supplement: Supplementary file 1 [file Data_Sheet_1.DOCX]

APPENDIX 1: FULL STIMULI LIST

Before including stimuli in our MEG recording for Study 2, they were submitted to the Amazon Mechanical Turk (AMT) interface. In addition to our MEG stimuli, we included a syntactically grammatical, but highly implausible condition for comparison. These sentences were constructed by switching the verb in the first clause with the verb in the inner relative clause on one-third of the sentences in each condition. For example, *The husband* nagged *the blankets and the wife who seldom* hogged *him did too.* Any individual items with consistently low ratings were considered for removal from the MEG experiment. As no extreme cases were found, all stimuli were included in MEG recordings. We gathered demographic information from 150 participants. Participants were removed from analysis if they were not a native speaker of English, had already completed the experiment (i.e., did it for a second time), far exceeded or fell below the average amount of time taken to complete the study, or had unreasonable data (e.g. the same response for all trials). Initially, 14 subjects were excluded based on these criteria, and replaced with 14 additional subjects. Items were distributed among 10 randomized different lists, so each list was completed by 15 subjects. Turk users saw each item and selected a plausibility rating on a 0 to 7 Likert scale (0=completely implausible).

| **Type** | **Interf.** | **Dependency** | **Sentence** | **Question** | **Response** |
| --- | --- | --- | --- | --- | --- |
| VPE | PAR | DEP | The husband hogged the blankets and the wife who seldom nagged him did too. | * |  |
| VPE | PAR | Control | The husband hogged the blankets and the wife who seldom nagged him did that too. | * |  |
| OR | PAR | DEP | The husband hogged the blankets that the wife who seldom nagged him grabbed afterward. | Did the wife grab the blankets? | Yes |
| OR | PAR | Control | The husband hogged the blankets and the wife who seldom nagged him grabbed them afterward. | * |  |
| RNR | PAR | DEP | The husband hogged and the wife who seldom nagged him grabbed the pillows. | * |  |
| RNR | PAR | Control | The husband hogged the sheets and the wife who seldom nagged him grabbed the pillows. | Did the husband hog the sheets? | Yes |
| FILL | PAR | DEP | The husband was in the kitchen and the wife who seldom nagged him did laundry. | * |  |
| VPE | Non-PAR | DEP | The husband hogged the blankets and Jane did too. | * |  |
| VPE | Non-PAR | Control | The husband hogged the blankets and Jane did that too. | Did Jane hog the pillows? | No |
| OR | Non-PAR | DEP | The husband hogged the blankets that Jane grabbed afterward. | * |  |
| OR | Non-PAR | Control | The husband hogged the blankets and Jane grabbed them afterward. | * |  |
| RNR | Non-PAR | DEP | The husband hogged and Jane grabbed the pillows. | Did the husband grab pillows? | No |
| RNR | Non-PAR | Control | The husband hogged the sheets and Jane grabbed the pillows. | * |  |
| FILL | Non-PAR | DEP | The husband was in the kitchen and Jane did laundry. | * |  |
| VPE | PAR | DEP | The mechanic inspected the bikes and the assistant who always helped him did too. | Did the assistant always help? | Yes |
| VPE | PAR | Control | The mechanic inspected the bikes and the assistant who always helped him did that too. | * |  |
| OR | PAR | DEP | The mechanic inspected the bikes that the assistant who always helped him chained afterward. | * |  |
| OR | PAR | Control | The mechanic inspected the bikes and the assistant who always helped him chained them afterward. | Did the assistant chain the bikes? | Yes |
| RNR | PAR | DEP | The mechanic inspected and the assistant who always helped him chained the tires. | * |  |
| RNR | PAR | Control | The mechanic inspected the bikes and the assistant who always helped him chained the tires. | * |  |
| FILL | PAR | DEP | The mechanic was in the dealership and the assistant who always helped him did cleaning. | Did the mechanic do cleaning? | No |
| VPE | Non-PAR | DEP | The mechanic inspected the bikes and Ralph did too. | * |  |
| VPE | Non-PAR | Control | The mechanic inspected the bikes and Ralph did that too. | * |  |
| OR | Non-PAR | DEP | The mechanic inspected the bikes that Ralph chained afterward. | Did Ralph inspect the bikes? | No |
| OR | Non-PAR | Control | The mechanic inspected the bikes and Ralph chained them afterward. | * |  |
| RNR | Non-PAR | DEP | The mechanic inspected and Ralph chained the tires. | * |  |
| RNR | Non-PAR | Control | The mechanic inspected the bikes and Ralph chained the tires. | Did Ralph chain tires? | Yes |
| FILL | Non-PAR | DEP | The mechanic was in the dealership and Ralph did cleaning. | * |  |
| VPE | PAR | DEP | The jeweler appraised the bracelets and the apprentice who usually assisted her did too. | * |  |
| VPE | PAR | Control | The jeweler appraised the bracelets and the apprentice who usually assisted her did that too. | Did the apprentice usually assist her? | Yes |
| OR | PAR | DEP | The jeweler appraised the bracelets that the apprentice who usually assisted her polished afterward. | * |  |
| OR | PAR | Control | The jeweler appraised the bracelets and the apprentice who usually assisted her polished them afterward. | * |  |
| RNR | PAR | DEP | The jeweler appraised and the apprentice who usually assisted her polished the watches. | Did the jeweler polish watches? | No |
| RNR | PAR | Control | The jeweler appraised the bracelets and the apprentice who usually assisted her polished the watches. | * |  |
| FILL | PAR | DEP | The jeweler was in the store and the apprentice who usually assisted her did polishing. | * |  |
| VPE | Non-PAR | DEP | The jeweler appraised the bracelets and Stella did too. | Did the jeweler polish bracelets? | No |
| VPE | Non-PAR | Control | The jeweler appraised the bracelets and Stella did that too. | * |  |
| OR | Non-PAR | DEP | The jeweler appraised the bracelets that Stella polished afterward. | * |  |
| OR | Non-PAR | Control | The jeweler appraised the bracelets and Stella polished them afterward. | Did Stella polish the bracelets? | Yes |
| RNR | Non-PAR | DEP | The jeweler appraised and Stella polished the watches. | * |  |
| RNR | Non-PAR | Control | The jeweler appraised the bracelets and Stella polished the watches. | * |  |
| FILL | Non-PAR | DEP | The jeweler was in the store and Stella did polishing. | Did Stella do polishing? | Yes |
| VPE | PAR | DEP | The stewardesses fastened the seatbelts and the passengers who rarely stand up did too. | * |  |
| VPE | PAR | Control | The stewardesses fastened the seatbelts and the passengers who rarely stand up did that too. | * |  |
| OR | PAR | DEP | The stewardesses fastened the seatbelts that the passengers who rarely stand up unfastened afterward. | Did the passengers fasten the seatbelts? | No |
| OR | PAR | Control | The stewardesses fastened the seatbelts and the passengers who rarely stand up unfastened them afterward. | * |  |
| RNR | PAR | DEP | The stewardesses fastened and the passengers who rarely stand up unfastened the tray-tables. | * |  |
| RNR | PAR | Control | The stewardesses fastened the seatbelts and the passengers who rarely stand up unfastened the tray-tables. | Did the stewardesses rarely stand up? | No |
| FILL | PAR | DEP | The stewardesses were in the plane and the passengers who rarely stand up did crosswords. | * |  |
| VPE | Non-PAR | DEP | The stewardesses fastened the seatbelts and Larry did too. | * |  |
| VPE | Non-PAR | Control | The stewardesses fastened the seatbelts and Larry did that too. | Did Larry fasten the seatbelts? | Yes |
| OR | Non-PAR | DEP | The stewardesses fastened the seatbelts that Larry unfastened afterward. | * |  |
| OR | Non-PAR | Control | The stewardesses fastened the seatbelts and Larry unfastened them afterward. | * |  |
| RNR | Non-PAR | DEP | The stewardesses fastened and Larry unfastened the tray-tables. | Did the stewardesses fasten the tray-tables? | Yes |
| RNR | Non-PAR | Control | The stewardesses fastened the seatbelts and Larry unfastened the tray-tables. | * |  |
| FILL | Non-PAR | DEP | The stewardesses were in the plane and Larry did crosswords. | * |  |
| VPE | PAR | DEP | The fisherman weighed the marlins and the sailor who frequently steered boats did too. | Did the fisherman gut the marlins? | No |
| VPE | PAR | Control | The fisherman weighed the marlins and the sailor who frequently steered boats did that too. | * |  |
| OR | PAR | DEP | The fisherman weighed the marlins that the sailor who frequently steered boats gutted afterward. | * |  |
| OR | PAR | Control | The fisherman weighed the marlins and the sailor who frequently steered boats gutted them afterward. | Did the sailor weigh the marlins? | No |
| RNR | PAR | DEP | The fisherman weighed and the sailor who frequently steered boats gutted the trout. | * |  |
| RNR | PAR | Control | The fisherman weighed the marlins and the sailor who frequently steered boats gutted the trout. | * |  |
| FILL | PAR | DEP | The fisherman was in the boat and the sailor who frequently steered boats did navigation. | Did the sailor frequently steer boats? | Yes |
| VPE | Non-PAR | DEP | The fisherman weighed the marlins and Linda did too. | * |  |
| VPE | Non-PAR | Control | The fisherman weighed the marlins and Linda did that too. | * |  |
| OR | Non-PAR | DEP | The fisherman weighed the marlins that Linda gutted afterward. | Did the fisherman weigh the marlins? | Yes |
| OR | Non-PAR | Control | The fisherman weighed the marlins and Linda gutted them afterward. | * |  |
| RNR | Non-PAR | DEP | The fisherman weighed and Linda gutted the trout. | * |  |
| RNR | Non-PAR | Control | The fisherman weighed the marlins and Linda gutted the trout. | Did Linda weigh the marlins? | No |
| FILL | Non-PAR | DEP | The fisherman was in the boat and Linda did navigation. | * |  |
| VPE | PAR | DEP | The guard examined the fences and the neighbor who often worried needlessly did too. | * |  |
| VPE | PAR | Control | The guard examined the fences and the neighbor who often worried needlessly did that too. | Did the neighbor latch the fences? | No |
| OR | PAR | DEP | The guard examined the fences that the neighbor who often worried needlessly latched afterward. | * |  |
| OR | PAR | Control | The guard examined the fences and the neighbor who often worried needlessly latched them afterward. | * |  |
| RNR | PAR | DEP | The guard examined and the neighbor who often worries needlessly latched the gates. | Did the guard examine the gates? | Yes |
| RNR | PAR | Control | The guard examined the fences and the neighbor who often worried needlessly latched the gates. | * |  |
| FILL | PAR | DEP | The guard was in the booth and the neighbor who often worried needlessly did surveillance . | * |  |
| VPE | Non-PAR | DEP | The guard examined the fences and Walter did too. | Did Walter examine the fences? | Yes |
| VPE | Non-PAR | Control | The guard examined the fences and Walter did that too. | * |  |
| OR | Non-PAR | DEP | The guard examined the fences that Walter latched afterward. | * |  |
| OR | Non-PAR | Control | The guard examined the fences and Walter latched them afterward. | Did Walter examine the fences? | No |
| RNR | Non-PAR | DEP | The guard examined and Walter latched the gates. | * |  |
| RNR | Non-PAR | Control | The guard examined the fences and Walter latched the gates. | * |  |
| FILL | Non-PAR | DEP | The guard was in the booth and Walter did surveillance . | Did Walter latch the fences? | No |
| VPE | PAR | DEP | The manager surveyed the stores and the cashier who occasionally worked overtime did too. | * |  |
| VPE | PAR | Control | The manager surveyed the stores and the cashier who occasionally worked overtime did that too. | * |  |
| OR | PAR | DEP | The manager surveyed the stores that the cashier who occasionally worked overtime locked afterward. | Did the cashier occasionally work overtime? | Yes |
| OR | PAR | Control | The manager surveyed the stores and the cashier who occasionally worked overtime locked them afterward. | * |  |
| RNR | PAR | DEP | The manager surveyed and the cashier who occasionally worked overtime locked the stockrooms. | * |  |
| RNR | PAR | Control | The manager surveyed the stores and the cashier who occasionally worked overtime locked the stockrooms. | Did the manager survey the stores? | Yes |
| FILL | PAR | DEP | The manager was in the back and the cashier who occasionally worked overtime did transactions. | * |  |
| VPE | Non-PAR | DEP | The manager surveyed the stores and Olivia did too. | * |  |
| VPE | Non-PAR | Control | The manager surveyed the stores and Olivia did that too. | Did Olivia lock the stockrooms? | No |
| OR | Non-PAR | DEP | The manager surveyed the stores that Olivia locked afterward. | * |  |
| OR | Non-PAR | Control | The manager surveyed the stores and Olivia locked them afterward. | * |  |
| RNR | Non-PAR | DEP | The manager surveyed and Olivia locked the stockrooms. | Did the manager lock stockrooms? | No |
| RNR | Non-PAR | Control | The manager surveyed the stores and Olivia locked the stockrooms. | * |  |
| FILL | Non-PAR | DEP | The manager was in the back and Olivia did transactions. | * |  |
| VPE | PAR | DEP | The administrator forwarded the flyers and the organizer who constantly emailed everyone did too. | Did the organizer forward the flyers? | Yes |
| VPE | PAR | Control | The administrator forwarded the flyers and the organizer who constantly emailed everyone did that too. | * |  |
| OR | PAR | DEP | The administrator forwarded the flyers that the organizer who constantly emailed everyone posted afterward. | * |  |
| OR | PAR | Control | The administrator forwarded the flyers and the organizer who constantly emailed everyone posted them afterward. | Did the organizer constantly email everyone? | Yes |
| RNR | PAR | DEP | The administrator forwarded and the organizer who constantly emailed everyone posted the announcements. | * |  |
| RNR | PAR | Control | The administrator forwarded the flyers and the organizer who constantly emailed everyone posted the announcements. | * |  |
| FILL | PAR | DEP | The administrator was in the office and the organizer who constantly emailed everyone did taxes. | Did the organizer forward the flyers? | No |
| VPE | Non-PAR | DEP | The administrator forwarded the flyers and Peter did too. | * |  |
| VPE | Non-PAR | Control | The administrator forwarded the flyers and Peter did that too. | * |  |
| OR | Non-PAR | DEP | The administrator forwarded the flyers that Peter posted afterward. | Did Peter forward the flyers? | No |
| OR | Non-PAR | Control | The administrator forwarded the flyers and Peter posted them afterward. | * |  |
| RNR | Non-PAR | DEP | The administrator forwarded and Peter posted the announcements. | * |  |
| RNR | Non-PAR | Control | The administrator forwarded the flyers and Peter posted the announcements. | Did Peter post announcements? | Yes |
| FILL | Non-PAR | DEP | The administrator was in the office and Peter did taxes. | * |  |
| VPE | PAR | DEP | The secretary copied the packets and the teacher who sometimes tutored students did too. | * |  |
| VPE | PAR | Control | The secretary copied the packets and the teacher who sometimes tutored students did that too. | Did the teacher copy the packets? | Yes |
| OR | PAR | DEP | The secretary copied the packets that the teacher who sometimes tutored students stapled afterward. | * |  |
| OR | PAR | Control | The secretary copied the packets and the teacher who sometimes tutored students stapled them afterward. | * |  |
| RNR | PAR | DEP | The secretary copied and the teacher who sometimes tutored students stapled the tests. | Did the secretary sometimes tutor students? | No |
| RNR | PAR | Control | The secretary copied the packets and the teacher who sometimes tutored students stapled the tests. | * |  |
| FILL | PAR | DEP | The secretary was in the school and the teacher who sometimes tutored students did review. | * |  |
| VPE | Non-PAR | DEP | The secretary copied the packets and Astrid did too. | Did Astrid staple the packets? | No |
| VPE | Non-PAR | Control | The secretary copied the packets and Astrid did that too. | * |  |
| OR | Non-PAR | DEP | The secretary copied the packets that Astrid stapled afterward. | * |  |
| OR | Non-PAR | Control | The secretary copied the packets and Astrid stapled them afterward. | Did the secretary copy the packets? | Yes |
| RNR | Non-PAR | DEP | The secretary copied and Astrid stapled the tests. | * |  |
| RNR | Non-PAR | Control | The secretary copied the packets and Astrid stapled the tests. | * |  |
| FILL | Non-PAR | DEP | The secretary was in the school and Astrid did review. | Did Astrid do review? | Yes |
| VPE | PAR | DEP | The seamstress assessed the clothes and the drycleaner who ordinarily took measurements did too. | * |  |
| VPE | PAR | Control | The seamstress assessed the clothes and the drycleaner who ordinarily took measurements did that too. | * |  |
| OR | PAR | DEP | The seamstress assessed the clothes that the drycleaner who ordinarily took measurements stitched afterward. | Did the seamstress stitch the clothes? | No |
| OR | PAR | Control | The seamstress assessed the clothes and the drycleaner who ordinarily took measurements stitched them afterward. | * |  |
| RNR | PAR | DEP | The seamstress assessed and the drycleaner who ordinarily took measurements stitched the curtains. | * |  |
| RNR | PAR | Control | The seamstress assessed the clothes and the drycleaner who ordinarily took measurements stitched the curtains. | Did the drycleaner assess the clothes? | No |
| FILL | PAR | DEP | The seamstress was in the attic and the drycleaner who ordinarily took measurements did sewing. | * |  |
| VPE | Non-PAR | DEP | The seamstress assessed the clothes and Philip did too. | * |  |
| VPE | Non-PAR | Control | The seamstress assessed the clothes and Philip did that too. | Did Philip assess the clothes? | Yes |
| OR | Non-PAR | DEP | The seamstress assessed the clothes that Philip stitched afterward. | * |  |
| OR | Non-PAR | Control | The seamstress assessed the clothes and Philip stitched them afterward. | * |  |
| RNR | Non-PAR | DEP | The seamstress assessed and Philip stitched the curtains. | Did the seamstress assess curtains? | Yes |
| RNR | Non-PAR | Control | The seamstress assessed the clothes and Philip stitched the curtains. | * |  |
| FILL | Non-PAR | DEP | The seamstress was in the attic and Philip did sewing. | * |  |
| VPE | PAR | DEP | The mother wore the boots and the child who infrequently fell down did too. | Did the mother infrequently fall down? | No |
| VPE | PAR | Control | The mother wore the boots and the child who infrequently fell down did that too. | * |  |
| OR | PAR | DEP | The mother wore the boots that the child who infrequently fell down untied afterward. | * |  |
| OR | PAR | Control | The mother wore the boots and the child who infrequently fell down untied them afterward. | Did the mother untie the boots? | No |
| RNR | PAR | DEP | The mother wore and the child who infrequently fell down untied the laces. | * |  |
| RNR | PAR | Control | The mother wore the boots and the child who infrequently fell down untied the laces. | * |  |
| FILL | PAR | DEP | The mother was in the house and the child who infrequently fell down did handstands. | Did the child do handstands? | Yes |
| VPE | Non-PAR | DEP | The mother wore the boots and Billy did too. | * |  |
| VPE | Non-PAR | Control | The mother wore the boots and Billy did that too. | * |  |
| OR | Non-PAR | DEP | The mother wore the boots that Billy untied afterward. | Did the mother wear the boots? | Yes |
| OR | Non-PAR | Control | The mother wore the boots and Billy untied them afterward. | * |  |
| RNR | Non-PAR | DEP | The mother wore and Billy untied the laces. | * |  |
| RNR | Non-PAR | Control | The mother wore the boots and Billy untied the laces. | Did Billy wear the boots? | No |
| FILL | Non-PAR | DEP | The mother was in the house and Billy did handstands. | * |  |
| VPE | PAR | DEP | The traveler scrutinized the suitcases and the agent who normally checked luggage did too. | * |  |
| VPE | PAR | Control | The traveler scrutinized the suitcases and the agent who normally checked luggage did that too. | Did the traveler normally check luggage? | No |
| OR | PAR | DEP | The traveler scrutinized the suitcases that the agent who normally checked luggage unzipped afterward. | * |  |
| OR | PAR | Control | The traveler scrutinized the suitcases and the agent who normally checked luggage unzipped them afterward. | * |  |
| RNR | PAR | DEP | The traveler scrutinized and the agent who normally checked luggage unzipped the purses. | Did the agent unzip purses? | Yes |
| RNR | PAR | Control | The traveler scrutinized the suitcases and the agent who normally checked luggage unzipped the purses. | * |  |
| FILL | PAR | DEP | The traveler was in the train and the agent who normally checked luggage did reservations. | * |  |
| VPE | Non-PAR | DEP | The traveler scrutinized the suitcases and Maxine did too. | Did Maxine scrutinize the suitcases? | Yes |
| VPE | Non-PAR | Control | The traveler scrutinized the suitcases and Maxine did that too. | * |  |
| OR | Non-PAR | DEP | The traveler scrutinized the suitcases that Maxine unzipped afterward. | * |  |
| OR | Non-PAR | Control | The traveler scrutinized the suitcases and Maxine unzipped them afterward. | Did Maxine scrutinize the suitcases? | No |
| RNR | Non-PAR | DEP | The traveler scrutinized and Maxine unzipped the bags. | * |  |
| RNR | Non-PAR | Control | The traveler scrutinized the suitcases and Maxine unzipped the bags. | * |  |
| FILL | Non-PAR | DEP | The traveler was in the train and Maxine did reservations. | Did the traveler do reservations? | No |
| VPE | PAR | DEP | The woman listed the instructions and the man who generally aided her did too. | * |  |
| VPE | PAR | Control | The woman listed the instructions and the man who generally aided her did that too. | * |  |
| OR | PAR | DEP | The woman listed the instructions that the man who generally aided her followed afterward. | Did the woman list the instructions? | Yes |
| OR | PAR | Control | The woman listed the instructions and the man who generally aided her followed them afterward. | * |  |
| RNR | PAR | DEP | The woman listed and the man who generally aided her followed the precautions. | * |  |
| RNR | PAR | Control | The woman listed the instructions and the man who generally aided her followed the precautions. | Did the man generally aid? | Yes |
| FILL | PAR | DEP | The woman was in the garage and the man who generally aided her did raking. | * |  |
| VPE | Non-PAR | DEP | The woman listed the instructions and Andy did too. | * |  |
| VPE | Non-PAR | Control | The woman listed the instructions and Andy did that too. | Did Andy follow the precautions? | No |
| OR | Non-PAR | DEP | The woman listed The instructions that Andy followed afterward. | * |  |
| OR | Non-PAR | Control | The woman listed The instructions and Andy followed them afterward. | * |  |
| RNR | Non-PAR | DEP | The woman listed and Andy followed the precautions. | Did the woman follow precautions? | No |
| RNR | Non-PAR | Control | The woman listed The instructions and Andy followed the precautions. | * |  |
| FILL | Non-PAR | DEP | The woman was in the garage and Andy did raking. | * |  |
| VPE | PAR | DEP | The waitress chipped the pitchers and the chef who never cleaned up did too. | Did the chef chip the pitchers? | Yes |
| VPE | PAR | Control | The waitress chipped the pitchers and the chef who never cleaned up did that too. | * |  |
| OR | PAR | DEP | The waitress chipped the pitchers that the chef who never cleaned up glued afterward. | * |  |
| OR | PAR | Control | The waitress chipped the pitchers and the chef who never cleaned up glued them afterward. | Did the waitress chip the pitchers? | Yes |
| RNR | PAR | DEP | The waitress chipped and the chef who never cleaned up glued the plates. | * |  |
| RNR | PAR | Control | The waitress chipped the pitchers and the chef who never cleaned up glued the plates. | * |  |
| FILL | PAR | DEP | The waitress was in the restaurant and the chef who never cleaned up did dishes. | Did the chef ever clean up? | No |
| VPE | Non-PAR | DEP | The waitress chipped the pitchers and Sandra did too. | * |  |
| VPE | Non-PAR | Control | The waitress chipped the pitchers and Sandra did that too. | * |  |
| OR | Non-PAR | DEP | The waitress chipped The pitchers that Sandra repaired afterward. | Did Sandra chip the pitchers? | No |
| OR | Non-PAR | Control | The waitress chipped The pitchers and Sandra repaired them afterward. | * |  |
| RNR | Non-PAR | DEP | The waitress chipped and Sandra repaired the glasses. | * |  |
| RNR | Non-PAR | Control | The waitress chipped The pitchers and Sandra repaired the glasses. | Did Sandra repair glasses? | Yes |
| FILL | Non-PAR | DEP | The waitress was in the restaurant and Sandra did dishes. | * |  |
| VPE | PAR | DEP | The princess threatened the dragons and the hero who typically won battles did too. | * |  |
| VPE | PAR | Control | The princess threatened the dragons and the hero who typically won battles did that too. | Did the princess threaten the dragons? | Yes |
| OR | PAR | DEP | The princess threatened the dragons that the hero who typically won battles defeated afterward. | * |  |
| OR | PAR | Control | The princess threatened the dragons and the hero who typically won battles defeated them afterward. | * |  |
| RNR | PAR | DEP | The princess threatened and the hero who typically won battles defeated the pirates. | Did the hero threaten pirates? | No |
| RNR | PAR | Control | The princess threatened the dragons and the hero who typically won battles defeated the pirates. | * |  |
| FILL | PAR | DEP | The princess was in the castle and the hero who typically won battles did quests. | * |  |
| VPE | Non-PAR | DEP | The princess threatened the dragons and Bianca did too. | Did the princess defeat the dragons? | No |
| VPE | Non-PAR | Control | The princess threatened the dragons and Bianca did that too. | * |  |
| OR | Non-PAR | DEP | The princess threatened the dragons that Bianca defeated afterward. | * |  |
| OR | Non-PAR | Control | The princess threatened the dragons and Bianca defeated them afterward. | Did Bianca defeat the dragons? | Yes |
| RNR | Non-PAR | DEP | The princess threatened and Bianca defeated the pirates. | * |  |
| RNR | Non-PAR | Control | The princess threatened The dragons and Bianca defeated the pirates. | * |  |
| FILL | Non-PAR | DEP | The princess was in the castle and Bianca did quests. | Did Bianca do quests? | Yes |
| VPE | PAR | DEP | The villain wounded the knights and the witch who scarcely escaped alive did too. | * |  |
| VPE | PAR | Control | The villain wounded the knights and the witch who scarcely escaped alive did that too. | * |  |
| OR | PAR | DEP | The villain wounded the knights that the witch who scarcely escaped alive healed afterward. | Did the villain scarcely escape alive? | No |
| OR | PAR | Control | The villain wounded the knights and the witch who scarcely escaped alive healed them afterward. | * |  |
| RNR | PAR | DEP | The villain wounded and the witch who scarcely escaped alive healed the princesses. | * |  |
| RNR | PAR | Control | The villain wounded the knights and the witch who scarcely escaped alive healed the princesses. | Did the villain heal princesses? | No |
| FILL | PAR | DEP | The villain was in the forest and the witch who scarcely escaped alive did spells. | * |  |
| VPE | Non-PAR | DEP | The villain wounded the knights and Helga did too. | * |  |
| VPE | Non-PAR | Control | The villain wounded the knights and Helga did that too. | Did the villain wound the knights? | Yes |
| OR | Non-PAR | DEP | The villain wounded the knights that Helga healed afterward. | * |  |
| OR | Non-PAR | Control | The villain wounded the knights and Helga healed them afterward. | * |  |
| RNR | Non-PAR | DEP | The villain wounded and Helga healed the princesses. | Did Helga heal princesses? | Yes |
| RNR | Non-PAR | Control | The villain wounded The knights and Helga healed the princesses. | * |  |
| FILL | Non-PAR | DEP | The villain was in the forest and Helga did spells. | * |  |
| VPE | PAR | DEP | The principal reviewed the reports and the teenager who regularly failed tests did too. | Did the principal hide the reports? | No |
| VPE | PAR | Control | The principal reviewed the reports and the teenager who regularly failed tests did that too. | * |  |
| OR | PAR | DEP | The principal reviewed the reports that the teenager who regularly failed tests hid afterward. | * |  |
| OR | PAR | Control | The principal reviewed the reports and the teenager who regularly failed tests hid them afterward. | Did the principal regularly fail tests? | No |
| RNR | PAR | DEP | The principal reviewed and the teenager who regularly failed tests hid the grades. | * |  |
| RNR | PAR | Control | The principal reviewed the reports and the teenager who regularly failed tests hid the grades. | * |  |
| FILL | PAR | DEP | The principal was in the building and the teenager who regularly failed tests did homework. | Did the teenager do homework? | Yes |
| VPE | Non-PAR | DEP | The principal reviewed the reports and Damion did too. | * |  |
| VPE | Non-PAR | Control | The principal reviewed the reports and Damion did that too. | * |  |
| OR | Non-PAR | DEP | The principal reviewed the reports that Damion hid afterward. | Did the principal review the reports? | Yes |
| OR | Non-PAR | Control | The principal reviewed the reports and Damion hid them afterward. | * |  |
| RNR | Non-PAR | DEP | The principal reviewed and Damion hid the grades. | * |  |
| RNR | Non-PAR | Control | The principal reviewed The reports and Damion hid the grades. | Did Damion review the reports? | No |
| FILL | Non-PAR | DEP | The principal was in the building and Damion did homework. | * |  |
| VPE | PAR | DEP | The magician disregarded the balloons and the clown who unceasingly told jokes did too. | * |  |
| VPE | PAR | Control | The magician disregarded the balloons and the clown who unceasingly told jokes did that too. | Did the clown inflate the balloons? | No |
| OR | PAR | DEP | The magician disregarded the balloons that the clown who unceasingly told jokes inflated afterward. | * |  |
| OR | PAR | Control | The magician disregarded the balloons and the clown who unceasingly told jokes inflated them afterward. | * |  |
| RNR | PAR | DEP | The magician disregarded and the clown who unceasingly told jokes inflated the beach-balls. | Did the clown unceasingly tell jokes? | Yes |
| RNR | PAR | Control | The magician disregarded the balloons and the clown who unceasingly told jokes inflated the beach-balls. | * |  |
| FILL | PAR | DEP | The magician was in the theater and the clown who unceasingly told jokes did tricks. | * |  |
| VPE | Non-PAR | DEP | The magician disregarded the balloons and Jeremy did too. | Did the magician disregard the balloons? | Yes |
| VPE | Non-PAR | Control | The magician disregarded the balloons and Jeremy did that too. | * |  |
| OR | Non-PAR | DEP | The magician disregarded the balloons that Jeremy inflated afterward. | * |  |
| OR | Non-PAR | Control | The magician disregarded the balloons and Jeremy inflated them afterward. | Did Jeremy disregard the balloons? | No |
| RNR | Non-PAR | DEP | The magician disregarded and Jeremy inflated the beach-balls. | * |  |
| RNR | Non-PAR | Control | The magician disregarded The balloons and Jeremy inflated the beach-balls. | * |  |
| FILL | Non-PAR | DEP | The magician was in the theater and Jeremy did tricks. | Did the magician do tricks? | No |
| VPE | PAR | DEP | The athlete injured the rivals and the trainer who routinely treated sprains did too. | * |  |
| VPE | PAR | Control | The athlete injured the rivals and the trainer who routinely treated sprains did that too. | * |  |
| OR | PAR | DEP | The athlete injured the rivals that the trainer who routinely treated sprains rehabilitated afterward. | Did the trainer rehabilitate the rivals? | Yes |
| OR | PAR | Control | The athlete injured the rivals and the trainer who routinely treated sprains rehabilitated them afterward. | * |  |
| RNR | PAR | DEP | The athlete injured and the trainer who routinely treats sprains rehabilitated the teammates. | * |  |
| RNR | PAR | Control | The athlete injured the rivals and the trainer who routinely treated sprains rehabilitated the teammates. | Did the athlete injure the rivals? | Yes |
| FILL | PAR | DEP | The athlete was in the gym and the trainer who routinely treated sprains did aerobics. | * |  |
| VPE | Non-PAR | DEP | The athlete injured the rivals and Kathryn did too. | * |  |
| VPE | Non-PAR | Control | The athlete injured the rivals and Kathryn did that too. | Did Kathryn rehabilitate the teammates? | No |
| OR | Non-PAR | DEP | The athlete injured the rivals that Kathryn rehabilitated afterward. | * |  |
| OR | Non-PAR | Control | The athlete injured the rivals and Kathryn rehabilitated them afterward. | * |  |
| RNR | Non-PAR | DEP | The athlete injured and Kathryn rehabilitated the teammates. | Did the athlete rehabilitate teammates? | No |
| RNR | Non-PAR | Control | The athlete injured The rivals and Kathryn rehabilitated the teammates. | * |  |
| FILL | Non-PAR | DEP | The athlete was in the gym and Kathryn did aerobics. | * |  |
| VPE | PAR | DEP | The senator supported the bills and the congressman who invariably gave speeches did too. | Did the congressman invariably give speeches? | Yes |
| VPE | PAR | Control | The senator supported the bills and the congressman who invariably gave speeches did that too. | * |  |
| OR | PAR | DEP | The senator supported the bills that the congressman who invariably gave speeches vetoed afterward. | * |  |
| OR | PAR | Control | The senator supported the bills and the congressman who invariably gave speeches vetoed them afterward. | Did the senator support the bills? | Yes |
| RNR | PAR | DEP | The senator supported and the congressman who invariably gave speeches vetoed the amendments. | * |  |
| RNR | PAR | Control | The senator supported the bills and the congressman who invariably gave speeches vetoed the amendments. | * |  |
| FILL | PAR | DEP | The senator was in the capitol and the congressman who invariably gave speeches did preparations. | Did the senator do preparations? | No |
| VPE | Non-PAR | DEP | The senator supported the bills and Dwight did too. | * |  |
| VPE | Non-PAR | Control | The senator supported the bills and Dwight did that too. | * |  |
| OR | Non-PAR | DEP | The senator supported the bills that Dwight vetoed afterward. | Did Dwight support the bills? | No |
| OR | Non-PAR | Control | The senator supported the bills and Dwight vetoed them afterward. | * |  |
| RNR | Non-PAR | DEP | The senator supported and Dwight vetoed the amendments. | * |  |
| RNR | Non-PAR | Control | The senator supported The bills and Dwight vetoed the amendments. | Did Dwight veto amendments? | Yes |
| FILL | Non-PAR | DEP | The senator was in the capitol and Dwight did preparations. | * |  |
| VPE | PAR | DEP | The clerk displayed the mannequins and the owner who repeatedly made changes did too. | * |  |
| VPE | PAR | Control | The clerk displayed the mannequins and the owner who repeatedly made changes did that too. | Did the owner repeatedly make changes? | Yes |
| OR | PAR | DEP | The clerk displayed the mannequins that the owner who repeatedly made changes removed afterward. | * |  |
| OR | PAR | Control | The clerk displayed the mannequins and the owner who repeatedly made changes removed them afterward. | * |  |
| RNR | PAR | DEP | The clerk displayed and the owner who repeatedly made changes removed the prices. | Did the clerk remove prices? | No |
| RNR | PAR | Control | The clerk displayed the mannequins and the owner who repeatedly made changes removed the prices. | * |  |
| FILL | PAR | DEP | The clerk was in the stockroom and the owner who repeatedly made changes did paperwork. | * |  |
| VPE | Non-PAR | DEP | The clerk displayed the mannequins and Angela did too. | Did Angela remove prices? | No |
| VPE | Non-PAR | Control | The clerk displayed the mannequins and Angela did that too. | * |  |
| OR | Non-PAR | DEP | The clerk displayed the mannequins that Angela removed afterward. | * |  |
| OR | Non-PAR | Control | The clerk displayed the mannequins and Angela removed them afterward. | Did Angela remove the mannequins? | Yes |
| RNR | Non-PAR | DEP | The clerk displayed and Angela removed the prices. | * |  |
| RNR | Non-PAR | Control | The clerk displayed The mannequins and Angela removed the prices. | * |  |
| FILL | Non-PAR | DEP | The clerk was in the stockroom and Angela did paperwork. | Did Angela do paperwork? | Yes |
| VPE | PAR | DEP | The decorator viewed the bedrooms and the realtor who consistently sold property did too. | * |  |
| VPE | PAR | Control | The decorator viewed the bedrooms and the realtor who consistently sold property did that too. | * |  |
| OR | PAR | DEP | The decorator viewed the bedrooms that the realtor who consistently sold property furnished afterward. | Did the realtor view the bedrooms? | No |
| OR | PAR | Control | The decorator viewed the bedrooms and the realtor who consistently sold property furnished them afterward. | * |  |
| RNR | PAR | DEP | The decorator viewed and the realtor who consistently sold property furnished the basements. | * |  |
| RNR | PAR | Control | The decorator viewed the bedrooms and the realtor who consistently sold property furnished the basements. | Did the decorator consistently sell property? | No |
| FILL | PAR | DEP | The decorator was in the den and the realtor who consistently sold property did calculations. | * |  |
| VPE | Non-PAR | DEP | The decorator viewed the bedrooms and Winston did too. | * |  |
| VPE | Non-PAR | Control | The decorator viewed the bedrooms and Winston did that too. | Did the decorator view the bedrooms? | Yes |
| OR | Non-PAR | DEP | The decorator viewed the bedrooms that Winston furnished afterward. | * |  |
| OR | Non-PAR | Control | The decorator viewed the bedrooms and Winston furnished them afterward. | * |  |
| RNR | Non-PAR | DEP | The decorator viewed and Winston furnished the basements. | Did Winston furnish basements? | Yes |
| RNR | Non-PAR | Control | The decorator viewed The bedrooms and Winston furnished the basements. | * |  |
| FILL | Non-PAR | DEP | The decorator was in the den and Winston did calculations. | * |  |
| VPE | PAR | DEP | The contractor adjusted the cabinets and the carpenter who persistently overcharged customers did too. | Did the carpenter sand the cabinets? | No |
| VPE | PAR | Control | The contractor adjusted the cabinets and the carpenter who persistently overcharged customers did that too. | * |  |
| OR | PAR | DEP | The contractor adjusted the cabinets that the carpenter who persistently overcharged customers sanded afterward. | * |  |
| OR | PAR | Control | The contractor adjusted the cabinets and the carpenter who persistently overcharged customers sanded them afterward. | Did the contractor sand the cabinets? | No |
| RNR | PAR | DEP | The contractor adjusted and the carpenter who persistently overcharged customers sanded the shelves. | * |  |
| RNR | PAR | Control | The contractor adjusted the cabinets and the carpenter who persistently overcharged customers sanded the shelves. | * |  |
| FILL | PAR | DEP | The contractor was in the basement and the carpenter who persistently overcharged customers did work. | Did the carpenter persistently overcharge customers? | Yes |
| VPE | Non-PAR | DEP | The contractor adjusted the cabinets and Pam did too. | * |  |
| VPE | Non-PAR | Control | The contractor adjusted the cabinets and Pam did that too. | * |  |
| OR | Non-PAR | DEP | The contractor adjusted the cabinets that Pam sanded afterward. | Did the contractor adjust the cabinets? | Yes |
| OR | Non-PAR | Control | The contractor adjusted the cabinets and Pam sanded them afterward. | * |  |
| RNR | Non-PAR | DEP | The contractor adjusted and Pam sanded the shelves. | * |  |
| RNR | Non-PAR | Control | The contractor adjusted The cabinets and Pam sanded the shelves. | Did Pam adjust the cabinets? | No |
| FILL | Non-PAR | DEP | The contractor was in the basement and Pam did work. | * |  |
| VPE | PAR | DEP | The preschooler built the towers and the babysitter who eternally awaited parents did too. | * |  |
| VPE | PAR | Control | The preschooler built the towers and the babysitter who eternally awaited parents did that too. | Did the babysitter disassemble the towers? | No |
| OR | PAR | DEP | The preschooler built the towers that the babysitter who eternally awaited parents disassembled afterward. | * |  |
| OR | PAR | Control | The preschooler built the towers and the babysitter who eternally awaited parents disassembled them afterward. | * |  |
| RNR | PAR | DEP | The preschooler built and the babysitter who eternally awaited parents disassembled the bridges. | Did the preschooler build bridges? | Yes |
| RNR | PAR | Control | The preschooler built the towers and the babysitter who eternally awaited parents disassembled the bridges. | * |  |
| FILL | PAR | DEP | The preschooler was in the playroom and the babysitter who eternally awaited parents did puzzles. | * |  |
| VPE | Non-PAR | DEP | The preschooler built the towers and Nick did too. | Did Nick build towers? | Yes |
| VPE | Non-PAR | Control | The preschooler built the towers and Nick did that too. | * |  |
| OR | Non-PAR | DEP | The preschooler built the towers that Nick disassembled afterward. | * |  |
| OR | Non-PAR | Control | The preschooler built the towers and Nick disassembled them afterward. | Did Nick build the towers? | No |
| RNR | Non-PAR | DEP | The preschooler built and Nick disassembled the bridges. | * |  |
| RNR | Non-PAR | Control | The preschooler built The towers and Nick disassembled the bridges. | * |  |
| FILL | Non-PAR | DEP | The preschooler was in the playroom and Nick did puzzles. | Did the preschooler do puzzles? | No |
| VPE | PAR | DEP | The boss enforced the policies and the employee who recurrently disobeyed rules did too. | * |  |
| VPE | PAR | Control | The boss enforced the policies and the employee who recurrently disobeyed rules did that too. | * |  |
| OR | PAR | DEP | The boss enforced the policies that the employee who recurrently disobeyed rules violated afterward. | Did the employee recurrently disobey rules? | Yes |
| OR | PAR | Control | The boss enforced the policies and the employee who recurrently disobeyed rules violated them afterward. | * |  |
| RNR | PAR | DEP | The boss enforced and the employee who recurrently disobeyed rules violated the dress-code. | * |  |
| RNR | PAR | Control | The boss enforced the policies and the employee who recurrently disobeyed rules violated the dress-code. | Did the employee violate dress-code? | Yes |
| FILL | PAR | DEP | The boss was in the cubicle and the employee who recurrently disobeyed rules did tasks. | * |  |
| VPE | Non-PAR | DEP | The boss enforced the policies and Jess did too. | * |  |
| VPE | Non-PAR | Control | The boss enforced the policies and Jess did that too. | Did Jess violate the dress-code? | No |
| OR | Non-PAR | DEP | The boss enforced the policies that Jess violated afterward. | * |  |
| OR | Non-PAR | Control | The boss enforced the policies and Jess violated them afterward. | * |  |
| RNR | Non-PAR | DEP | The boss enforced and Jess violated the dress-code. | Did the boss violate dress-code? | No |
| RNR | Non-PAR | Control | The boss enforced the policies and Jess violated the dress-code. | * |  |
| FILL | Non-PAR | DEP | The boss was in the cubicle and Jess did tasks. | * |  |
| VPE | PAR | DEP | The criminal dismissed the weapons and the policeman who perpetually investigated crimes did too. | Did the criminal dismiss the weapons? | Yes |
| VPE | PAR | Control | The criminal dismissed the weapons and the policeman who perpetually investigated crimes did that too. | * |  |
| OR | PAR | DEP | The criminal dismissed the weapons that the policeman who perpetually investigated crimes uncovered afterward. | * |  |
| OR | PAR | Control | The criminal dismissed the weapons and the policeman who perpetually investigated crimes uncovered them afterward. | Did the policeman perpetually investigate crimes? | Yes |
| RNR | PAR | DEP | The criminal dismissed and the policeman who perpetually investigated crimes uncovered the evidence. | * |  |
| RNR | PAR | Control | The criminal dismissed the weapons and the policeman who perpetually investigated crimes uncovered the evidence. | * |  |
| FILL | PAR | DEP | The criminal was in the jail and the policeman who perpetually investigated crimes did questioning. | Did the policeman dismiss the weapons? | No |
| VPE | Non-PAR | DEP | The criminal dismissed the weapons and Toby did too. | * |  |
| VPE | Non-PAR | Control | The criminal dismissed the weapons and Toby did that too. | * |  |
| OR | Non-PAR | DEP | The criminal dismissed the weapons that Toby uncovered afterward. | Did Toby dismiss the weapons? | No |
| OR | Non-PAR | Control | The criminal dismissed the weapons and Toby uncovered them afterward. | * |  |
| RNR | Non-PAR | DEP | The criminal dismissed and Toby uncovered the evidence. | * |  |
| RNR | Non-PAR | Control | The criminal dismissed the weapons and Toby uncovered the evidence. | Did Toby uncover evidence? | Yes |
| FILL | Non-PAR | DEP | The criminal was in the jail and Toby did questioning. | * |  |
| VPE | PAR | DEP | The caddy compared the clubs and the golfer who intermittently missed shots did too. | * |  |
| VPE | PAR | Control | The caddy compared the clubs and the golfer who intermittently missed shots did that too. | Did the golfer compare the clubs? | Yes |
| OR | PAR | DEP | The caddy compared the clubs that the golfer who intermittently missed shots used afterward. | * |  |
| OR | PAR | Control | The caddy compared the clubs and the golfer who intermittently missed shots used them afterward. | * |  |
| RNR | PAR | DEP | The caddy compared and the golfer who intermittently missed shots used the tees. | Did the caddy intermittently miss shots? | No |
| RNR | PAR | Control | The caddy compared the clubs and the golfer who intermittently missed shots used the tees. | * |  |
| FILL | PAR | DEP | The caddy was in the cart and the golfer who intermittently missed shots did practice. | * |  |
| VPE | Non-PAR | DEP | The caddy compared the clubs and Phyllis did too. | Did Phyllis compare tees? | No |
| VPE | Non-PAR | Control | The caddy compared the clubs and Phyllis did that too. | * |  |
| OR | Non-PAR | DEP | The caddy compared the clubs that Phyllis used afterward. | * |  |
| OR | Non-PAR | Control | The caddy compared the clubs and Phyllis used them afterward. | Did the caddy compare the clubs? | Yes |
| RNR | Non-PAR | DEP | The caddy compared and Phyllis used the tees. | * |  |
| RNR | Non-PAR | Control | The caddy compared the clubs and Phyllis used the tees. | * |  |
| FILL | Non-PAR | DEP | The caddy was in the cart and Phyllis did practice. | Did Phyllis do practice? | Yes |
| VPE | PAR | DEP | The navigator mapped the destinations and the driver who periodically got lost did too. | * |  |
| VPE | PAR | Control | The navigator mapped the destinations and the driver who periodically got lost did that too. | * |  |
| OR | PAR | DEP | The navigator mapped the destinations that the driver who periodically got lost found afterward. | Did the driver map the destinations? | No |
| OR | PAR | Control | The navigator mapped the destinations and the driver who periodically got lost found them afterward. | * |  |
| RNR | PAR | DEP | The navigator mapped and the driver who periodically got lost found the gasoline. | * |  |
| RNR | PAR | Control | The navigator mapped the destinations and the driver who periodically got lost found the gasoline. | Did the navigator find gasoline? | No |
| FILL | PAR | DEP | The navigator was in the car and the driver who periodically got lost did turns. | * |  |
| VPE | Non-PAR | DEP | The navigator mapped the destinations and Jim did too. | * |  |
| VPE | Non-PAR | Control | The navigator mapped the destinations and Jim did that too. | Did the navigator map the destinations? | Yes |
| OR | Non-PAR | DEP | The navigator mapped the destinations that Jim found afterward. | * |  |
| OR | Non-PAR | Control | The navigator mapped the destinations and Jim found them afterward. | * |  |
| RNR | Non-PAR | DEP | The navigator mapped and Jim found the gasoline. | Did Jim Jim find gasoline? | Yes |
| RNR | Non-PAR | Control | The navigator mapped the destinations and Jim found the gasoline. | * |  |
| FILL | Non-PAR | DEP | The navigator was in the car and Jim did turns. | * |  |
| VPE | PAR | DEP | The repairman tightened the pipes and the plumber who mostly made errors did too. | Did the repairman mostly make errors? | No |
| VPE | PAR | Control | The repairman tightened the pipes and the plumber who mostly made errors did that too. | * |  |
| OR | PAR | DEP | The repairman tightened the pipes that the plumber who mostly made errors broke afterward. | * |  |
| OR | PAR | Control | The repairman tightened the pipes and the plumber who mostly made error broke them afterward. | Did the plumber tighten the pipes? | No |
| RNR | PAR | DEP | The repairman tightened and the plumber who mostly made errors broke the sinks. | * |  |
| RNR | PAR | Control | The repairman tightened the pipes and the plumber who mostly made errors broke the sinks. | * |  |
| FILL | PAR | DEP | The repairman was in the bathroom and the plumber who mostly made errors did damage. | Did the plumber do damage? | Yes |
| VPE | Non-PAR | DEP | The repairman tightened the pipes and Elena did too. | * |  |
| VPE | Non-PAR | Control | The repairman tightened the pipes and Elena did that too. | * |  |
| OR | Non-PAR | DEP | The repairman tightened the pipes that Elena broke afterward. | Did the repairman tighten the pipes? | Yes |
| OR | Non-PAR | Control | The repairman tightened the pipes and Elena broke them afterward. | * |  |
| RNR | Non-PAR | DEP | The repairman tightened and Elena broke the sinks. | * |  |
| RNR | Non-PAR | Control | The repairman tightened the pipes and Elena broke the sinks. | Did Elena tighten the pipes? | No |
| FILL | Non-PAR | DEP | The repairman was in the bathroom and Elena did damage. | * |  |
| VPE | PAR | DEP | The sister tasted the fries and the brother who subsequently fell asleep did too. | * |  |
| VPE | PAR | Control | The sister tasted the fries and the brother who subsequently fell asleep did that too. | Did the sister subsequently fall asleep? | No |
| OR | PAR | DEP | The sister tasted the fries that the brother who subsequently fell asleep salted afterward. | * |  |
| OR | PAR | Control | The sister tasted the fries and the brother who subsequently fell asleep salted them afterward. | * |  |
| RNR | PAR | DEP | The sister tasted and the brother who subsequently fell asleep salted the pasta. | Did the sister taste pasta? | Yes |
| RNR | PAR | Control | The sister tasted the fries and the brother who subsequently fell asleep salted the pasta. | * |  |
| FILL | PAR | DEP | The sister was in the pool and the brother who subsequently fell asleep did laps. | * |  |
| VPE | Non-PAR | DEP | The sister tasted the fries and Seth did too. | Did Seth taste the fries? | Yes |
| VPE | Non-PAR | Control | The sister tasted the fries and Seth did that too. | * |  |
| OR | Non-PAR | DEP | The sister tasted the fries that Seth salted afterward. | * |  |
| OR | Non-PAR | Control | The sister tasted the fries and Seth salted them afterward. | Did Seth taste the fries? | No |
| RNR | Non-PAR | DEP | The sister tasted and Seth salted the pasta. | * |  |
| RNR | Non-PAR | Control | The sister tasted the fries and Seth salted the pasta. | * |  |
| FILL | Non-PAR | DEP | The sister was in the pool and Seth did laps. | Did Seth salt the fries? | No |
| VPE | PAR | DEP | The CEO praised the workers and the politician who ultimately gained votes did too. | * |  |
| VPE | PAR | Control | The CEO praised the workers and the politician who ultimately gained votes did that too. | * |  |
| OR | PAR | DEP | The CEO praised the workers that the politician who ultimately gained votes criticized afterward. | Did the CEO praise the workers? | Yes |
| OR | PAR | Control | The CEO praised the workers and the politician who ultimately gained votes criticized them afterward. | * |  |
| RNR | PAR | DEP | The CEO praised and the politician who ultimately gained votes criticized the consumers. | * |  |
| RNR | PAR | Control | The CEO praised the workers and the politician who ultimately gained votes criticized the consumers. | Did the politician ultimately gain votes? | Yes |
| FILL | PAR | DEP | The CEO was in the boardroom and the politician who ultimately gained votes did interviews. | * |  |
| VPE | Non-PAR | DEP | The CEO praised the workers and Cecelia did too. | * |  |
| VPE | Non-PAR | Control | The CEO praised the workers and Cecelia did that too. | Did Cecelia criticize the consumers? | No |
| OR | Non-PAR | DEP | The CEO praised the workers that Cecelia criticized afterward. | * |  |
| OR | Non-PAR | Control | The CEO praised the workers and Cecelia criticized them afterward. | * |  |
| RNR | Non-PAR | DEP | The CEO praised and Cecelia criticized the consumers. | Did Cecelia praise consumers? | No |
| RNR | Non-PAR | Control | The CEO praised the workers and Cecelia criticized the consumers. | * |  |
| FILL | Non-PAR | DEP | The CEO was in the boardroom and Cecelia did interviews. | * |  |
| VPE | PAR | DEP | The researcher checked the tubes and the doctor who primarily ran experiments did too. | Did the researcher check the tubes? | Yes |
| VPE | PAR | Control | The researcher checked the tubes and the doctor who primarily ran experiments did that too. | * |  |
| OR | PAR | DEP | The researcher checked the tubes that the doctor who primarily ran experiments adjusted afterward. | * |  |
| OR | PAR | Control | The researcher checked the tubes and the doctor who primarily ran experiments adjusted them afterward. | Did the doctor adjust the tubes? | Yes |
| RNR | PAR | DEP | The researcher checked and the doctor who primarily ran experiments adjusted the oxygen. | * |  |
| RNR | PAR | Control | The researcher checked the tubes and the doctor who primarily ran experiments adjusted the oxygen. | * |  |
| FILL | PAR | DEP | The researcher was in the lab and the doctor who primarily ran experiments did tests. | Did the researcher primarily run experiments? | No |
| VPE | Non-PAR | DEP | The researcher checked the tubes and David did too. | * |  |
| VPE | Non-PAR | Control | The researcher checked the tubes and David did that too. | * |  |
| OR | Non-PAR | DEP | The researcher checked the tubes that David adjusted afterward. | Did David check the tubes? | No |
| OR | Non-PAR | Control | The researcher checked the tubes and David adjusted them afterward. | * |  |
| RNR | Non-PAR | DEP | The researcher checked and David adjusted the oxygen. | * |  |
| RNR | Non-PAR | Control | The researcher checked the tubes and David adjusted the oxygen. | Did David adjust oxygen? | Yes |
| FILL | Non-PAR | DEP | The researcher was in the lab and David did tests. | * |  |
| VPE | PAR | DEP | The tenant tested the bulbs and the electrician who commonly carried flashlights did too. | * |  |
| VPE | PAR | Control | The tenant tested the bulbs and the electrician who commonly carried flashlights did that too. | Did the electrician test the bulbs? | Yes |
| OR | PAR | DEP | The tenant tested the bulbs that the electrician who commonly carried flashlights replaced afterward. | * |  |
| OR | PAR | Control | The tenant tested the bulbs and the electrician who commonly carried flashlights replaced them afterward. | * |  |
| RNR | PAR | DEP | The tenant tested and the electrician who commonly carried flashlights replaced the switches. | Did the tenant replace switches? | No |
| RNR | PAR | Control | The tenant tested the bulbs and the electrician who commonly carried flashlights replaced the switches. | * |  |
| FILL | PAR | DEP | The tenant was in the apartment and the electrician who commonly carried flashlights did wiring. | * |  |
| VPE | Non-PAR | DEP | The tenant tested the bulbs and Georgia did too. | Did the tenant replace the bulbs? | No |
| VPE | Non-PAR | Control | The tenant tested the bulbs and Georgia did that too. | * |  |
| OR | Non-PAR | DEP | The tenant tested the bulbs that Georgia replaced afterward. | * |  |
| OR | Non-PAR | Control | The tenant tested the bulbs and Georgia replaced them afterward. | Did Georgia replace the bulbs? | Yes |
| RNR | Non-PAR | DEP | The tenant tested and Georgia replaced the switches. | * |  |
| RNR | Non-PAR | Control | The tenant tested the bulbs and Georgia replaced the switches. | * |  |
| FILL | Non-PAR | DEP | The tenant was in the apartment and Georgia did wiring. | Did Georgia do wiring? | Yes |
| VPE | PAR | DEP | The florist promoted the bouquets and the bridesmaid who traditionally chose flowers did too. | * |  |
| VPE | PAR | Control | The florist promoted the bouquets and the bridesmaid who traditionally chose flowers did that too. | * |  |
| OR | PAR | DEP | The florist promoted the bouquets that the bridesmaid who traditionally chose flowers purchased afterward. | Did the florist traditionally choose flowers? | No |
| OR | PAR | Control | The florist promoted the bouquets and the bridesmaid who traditionally chose flowers purchased them afterward. | * |  |
| RNR | PAR | DEP | The florist promoted and the bridesmaid who traditionally chose flowers purchased the centerpieces. | * |  |
| RNR | PAR | Control | The florist promoted the bouquets and the bridesmaid who traditionally chose flowers purchased the centerpieces. | Did the florist purchase centerpieces? | No |
| FILL | PAR | DEP | The florist was in the venue and the bridesmaid who traditionally chose flowers did hair. | * |  |
| VPE | Non-PAR | DEP | The florist promoted the bouquets and Brett did too. | * |  |
| VPE | Non-PAR | Control | The florist promoted the bouquets and Brett did that too. | Did the florist promote the bouquets? | Yes |
| OR | Non-PAR | DEP | The florist promoted the bouquets that Brett purchased afterward. | * |  |
| OR | Non-PAR | Control | The florist promoted the bouquets and Brett purchased them afterward. | * |  |
| RNR | Non-PAR | DEP | The florist promoted and Brett purchased the centerpieces. | Did Brett purchase centerpieces? | Yes |
| RNR | Non-PAR | Control | The florist promoted the bouquets and Brett purchased the centerpieces. | * |  |
| FILL | Non-PAR | DEP | The florist was in the venue and Brett did hair. | * |  |
| VPE | PAR | DEP | The curator valued the stamps and the historian who habitually collected artifacts did too. | Did the historian analyze the stamps? | No |
| VPE | PAR | Control | The curator valued the stamps and the historian who habitually collected artifacts did that too. | * |  |
| OR | PAR | DEP | The curator valued the stamps that the historian who habitually collected artifacts analyzed afterward. | * |  |
| OR | PAR | Control | The curator valued the stamps and the historian who habitually collected artifacts analyzed them afterward. | Did the curator habitually collect artifacts? | No |
| RNR | PAR | DEP | The curator valued and the historian who habitually collected artifacts analyzed the paintings. | * |  |
| RNR | PAR | Control | The curator valued the stamps and the historian who habitually collected artifacts analyzed the paintings. | * |  |
| FILL | PAR | DEP | The curator was in the museum and the historian who habitually collected artifacts did research. | Did the historian do research? | Yes |
| VPE | Non-PAR | DEP | The curator valued the stamps and Caroline did too. | * |  |
| VPE | Non-PAR | Control | The curator valued the stamps and Caroline did that too. | * |  |
| OR | Non-PAR | DEP | The curator valued the stamps that Caroline analyzed afterward. | Did the curator value the stamps? | Yes |
| OR | Non-PAR | Control | The curator valued the stamps and Caroline analyzed them afterward. | * |  |
| RNR | Non-PAR | DEP | The curator valued and Caroline analyzed the paintings. | * |  |
| RNR | Non-PAR | Control | The curator valued the stamps and Caroline analyzed the paintings. | Did Caroline value the stamps? | No |
| FILL | Non-PAR | DEP | The curator was in the museum and Caroline did research. | * |  |
| VPE | PAR | DEP | The city accessed the parks and the volunteers who finally finished planting did too. | * |  |
| VPE | PAR | Control | The city accessed the parks and the volunteers who finally finished planting did that too. | Did the city maintain the parks? | No |
| OR | PAR | DEP | The city accessed the parks that the volunteers who finally finished planting maintained afterward. | * |  |
| OR | PAR | Control | The city accessed the parks and the volunteers who finally finished planting maintained them afterward. | * |  |
| RNR | PAR | DEP | The city accessed and the volunteers who finally finished planting maintained the gardens. | Did the volunteers finally finish planting? | Yes |
| RNR | PAR | Control | The city accessed the parks and the volunteers who finally finished maintained the gardens. | * |  |
| FILL | PAR | DEP | The city was in the South and the volunteers who finally finished planting did fundraising. | * |  |
| VPE | Non-PAR | DEP | The city accessed the parks and Bryan did too. | Did the city access the parks? | Yes |
| VPE | Non-PAR | Control | The city accessed the parks and Bryan did that too. | * |  |
| OR | Non-PAR | DEP | The city accessed the parks that Bryan maintained afterward. | * |  |
| OR | Non-PAR | Control | The city accessed the parks and Bryan maintained them afterward. | Did Bryan access the parks? | No |
| RNR | Non-PAR | DEP | The city accessed and Bryan maintained the gardens. | * |  |
| RNR | Non-PAR | Control | The city accessed the parks and Bryan maintained the gardens. | * |  |
| FILL | Non-PAR | DEP | The city was in the South and Bryan did fundraising. | Did the city do fundraising? | No |
| VPE | PAR | DEP | The landscaper watered the bushes and the groundskeeper who steadily pulled weeds did too. | * |  |
| VPE | PAR | Control | The landscaper watered the bushes and the groundskeeper who steadily pulled weeds did that too. | * |  |
| OR | PAR | DEP | The landscaper watered the bushes that the groundskeeper who steadily pulled weeds trimmed afterward. | Did the landscaper water the bushes? | Yes |
| OR | PAR | Control | The landscaper watered the bushes and the groundskeeper who steadily pulled weeds trimmed them afterward. | * |  |
| RNR | PAR | DEP | The landscaper watered and the groundskeeper who steadily pulled weeds trimmed the trees. | * |  |
| RNR | PAR | Control | The landscaper watered the bushes and the groundskeeper who steadily pulled weeds trimmed the trees. | Did the groundskeeper trim trees? | Yes |
| FILL | PAR | DEP | The landscaper was in the yard and the groundskeeper who steadily pulled weeds did gardening. | * |  |
| VPE | Non-PAR | DEP | The landscaper watered the bushes and Lauren did too. | * |  |
| VPE | Non-PAR | Control | The landscaper watered the bushes and Lauren did that too. | Did Lauren trim the trees? | No |
| OR | Non-PAR | DEP | The landscaper watered the bushes that Lauren trimmed afterward. | * |  |
| OR | Non-PAR | Control | The landscaper watered the bushes and Lauren trimmed them afterward. | * |  |
| RNR | Non-PAR | DEP | The landscaper watered and Lauren trimmed the trees. | Did the landscaper trims trees? | No |
| RNR | Non-PAR | Control | The landscaper watered the bushes and Lauren trimmed the trees. | * |  |
| FILL | Non-PAR | DEP | The landscaper was in the yard and Lauren did gardening. | * |  |
| VPE | PAR | DEP | The bookie predicted the bets and the gambler who briefly contemplated quitting did too. | Did the gambler briefly contemplated quitting? | Yes |
| VPE | PAR | Control | The bookie predicted the bets and the gambler who briefly contemplated quitting did that too. | * |  |
| OR | PAR | DEP | The bookie predicted the bets that the gambler who briefly contemplated quitting owed afterward. | * |  |
| OR | PAR | Control | The bookie predicted the bets and the gambler who briefly contemplated quitting owed them afterward. | Did the gambler owe the bets? | Yes |
| RNR | PAR | DEP | The bookie predicted and the gambler who briefly contemplated quitting owed the payments. | * |  |
| RNR | PAR | Control | The bookie predicted the bets and the gambler who briefly contemplated quitting owed the payments. | * |  |
| FILL | PAR | DEP | The bookie was in the casino and the gambler who briefly contemplated quitting did slots. | Did the bookie do slots? | No |
| VPE | Non-PAR | DEP | The bookie predicted the bets and Steven did too. | * |  |
| VPE | Non-PAR | Control | The bookie predicted the bets and Steven did that too. | * |  |
| OR | Non-PAR | DEP | The bookie predicted the bets that Steven owed afterward. | Did Steven predict the bets? | No |
| OR | Non-PAR | Control | The bookie predicted the bets and Steven owed them afterward. | * |  |
| RNR | Non-PAR | DEP | The bookie predicted and Steven owed the payments. | * |  |
| RNR | Non-PAR | Control | The bookie predicted the bets and Steven owed the payments. | Did Steven owe payments? | Yes |
| FILL | Non-PAR | DEP | The bookie was in the casino and Steven did slots. | * |  |
| VPE | PAR | DEP | The foundation requested the funds and the students who customarily accepted donations did too. | * |  |
| VPE | PAR | Control | The foundation requested the funds and the students who customarily accepted donations did that too. | Did the students customarily accept donations? | Yes |
| OR | PAR | DEP | The foundation requested the funds that the students who customarily accepted donations received afterward. | * |  |
| OR | PAR | Control | The foundation requested the funds and the students who customarily accepted donations received them afterward. | * |  |
| RNR | PAR | DEP | The foundation requested and the students who customarily accepted donations received the scholarships. | Did the students request scholarships? | No |
| RNR | PAR | Control | The foundation requested the funds and the students who customarily accepted donations received the scholarships. | * |  |
| FILL | PAR | DEP | The foundation was in the flood and the students who customarily accepted donations did volunteering. | * |  |
| VPE | Non-PAR | DEP | The foundation requested the funds and Anne did too. | Did Anne receive scholarships? | No |
| VPE | Non-PAR | Control | The foundation requested the funds and Anne did that too. | * |  |
| OR | Non-PAR | DEP | The foundation requested the funds that Anne received afterward. | * |  |
| OR | Non-PAR | Control | The foundation requested the funds and Anne received them afterward. | Did the students request the funds? | Yes |
| RNR | Non-PAR | DEP | The foundation requested and Anne received the scholarships. | * |  |
| RNR | Non-PAR | Control | The foundation requested the funds and Anne received the scholarships. | * |  |
| FILL | Non-PAR | DEP | The foundation was in the flood and Anne did volunteering. | Did Anne do volunteering? | Yes |
| VPE | PAR | DEP | The host recommended the speakers and the director who sporadically attended rehearsals did too. | * |  |
| VPE | PAR | Control | The host recommended the speakers and the director who sporadically attended rehearsals did that too. | * |  |
| OR | PAR | DEP | The host recommended the speakers that the director who sporadically attended rehearsals invited afterward. | Did the director recommend the speakers? | No |
| OR | PAR | Control | The host recommended the speakers and the director who sporadically attended rehearsals invited them afterward. | * |  |
| RNR | PAR | DEP | The host recommended and the director who sporadically attended rehearsals invited the staff. | * |  |
| RNR | PAR | Control | The host recommended the speakers and the director who sporadically attended rehearsals invited the staff. | Did the host sporadically attend rehearsals? | No |
| FILL | PAR | DEP | The host was in the seat and the director who sporadically attended rehearsals did filming. | * |  |
| VPE | Non-PAR | DEP | The host recommended the speakers and Owen did too. | * |  |
| VPE | Non-PAR | Control | The host recommended the speakers and Owen did that too. | Did the host recommend the speakers? | Yes |
| OR | Non-PAR | DEP | The host recommended the speakers that Owen invited afterward. | * |  |
| OR | Non-PAR | Control | The host recommended the speakers and Owen invited them afterward. | * |  |
| RNR | Non-PAR | DEP | The host recommended and Owen invited the staff. | Did the host recommend staff? | Yes |
| RNR | Non-PAR | Control | The host recommended the speakers and Owen invited the staff. | * |  |
| FILL | Non-PAR | DEP | The host was in the seat and Owen did filming. | * |  |
| VPE | PAR | DEP | The hiker avoided the dangers and the camper who mainly liked adventures did too. | Did the hiker face the dangers? | No |
| VPE | PAR | Control | The hiker avoided the dangers and the camper who mainly liked adventures did that too. | * |  |
| OR | PAR | DEP | The hiker avoided the dangers that the camper who mainly liked adventures faced afterward. | * |  |
| OR | PAR | Control | The hiker avoided the dangers and the camper who mainly liked adventures faced them afterward. | Did the camper avoid the dangers? | No |
| RNR | PAR | DEP | The hiker avoided and the camper who mainly liked adventures faced the cliffs. | * |  |
| RNR | PAR | Control | The hiker avoided the dangers and the camper who mainly liked adventures faced the cliffs. | * |  |
| FILL | PAR | DEP | The hiker was in the tent and the camper who mainly liked adventures did archery. | Did the camper mainly liked adventures? | Yes |
| VPE | Non-PAR | DEP | The hiker avoided the dangers and Bonnie did too. | * |  |
| VPE | Non-PAR | Control | The hiker avoided the dangers and Bonnie did that too. | * |  |
| OR | Non-PAR | DEP | The hiker avoided the dangers that Bonnie faced afterward. | Did the hiker avoid the dangers? | Yes |
| OR | Non-PAR | Control | The hiker avoided the dangers and Bonnie faced them afterward. | * |  |
| RNR | Non-PAR | DEP | The hiker avoided and Bonnie faced the cliffs. | * |  |
| RNR | Non-PAR | Control | The hiker avoided the dangers and Bonnie faced the cliffs. | Did the hiker face cliffs? | No |
| FILL | Non-PAR | DEP | The hiker was in the tent and Bonnie did archery. | * |  |
| VPE | PAR | DEP | The biker dented the cars and the valet who initially got tickets did too. | * |  |
| VPE | PAR | Control | The biker dented the cars and the valet who initially got tickets did that too. | Did the valet park the cars? | No |
| OR | PAR | DEP | The biker dented the cars that the valet who initially got tickets parked afterward. | * |  |
| OR | PAR | Control | The biker dented the cars and the valet who initially got tickets parked them afterward. | * |  |
| RNR | PAR | DEP | The biker dented and the valet who initially got tickets parked the trucks. | Did the biker dent trucks? | Yes |
| RNR | PAR | Control | The biker dented the car and the valet who initially got tickets parked the trucks. | * |  |
| FILL | PAR | DEP | The biker was in the lot and the valet who initially got tickets did parking. | * |  |
| VPE | Non-PAR | DEP | The biker dented the cars and Pat did too. | Did the biker dent the cars? | Yes |
| VPE | Non-PAR | Control | The biker dented the cars and Pat did that too. | * |  |
| OR | Non-PAR | DEP | The biker dented the cars that Pat parked afterward. | * |  |
| OR | Non-PAR | Control | The biker dented the cars and Pat parked them afterward. | Did Pat dent the cars? | No |
| RNR | Non-PAR | DEP | The biker dented and Pat parked the trucks. | * |  |
| RNR | Non-PAR | Control | The biker dented the car and Pat parked the trucks. | * |  |
| FILL | Non-PAR | DEP | The biker was in the lot and Pat did parking. | Did the biker do parking? | No |
| VPE | PAR | DEP | The hospital needed the medications and the pharmacy that principally delivered items did too. | * |  |
| VPE | PAR | Control | The hospital needed the medications and the pharmacy that principally delivered items did that too. | * |  |
| OR | PAR | DEP | The hospital needed the medications that the pharmacy that principally delivered items ordered afterward. | Did the pharmacy principally delivered items? | Yes |
| OR | PAR | Control | The hospital needed the medications and the pharmacy that principally delivered items ordered them afterward. | * |  |
| RNR | PAR | DEP | The hospital needed and the pharmacy that principally delivered items ordered the supplies. | * |  |
| RNR | PAR | Control | The hospital needed the medications and the pharmacy that principally delivered items ordered the supplies. | Did the hospital need the medications? | Yes |
| FILL | PAR | DEP | The hospital was in the suburbs and the pharmacy that principally delivered items did prescriptions. | * |  |
| VPE | Non-PAR | DEP | The hospital needed the medications and George did too. | * |  |
| VPE | Non-PAR | Control | The hospital needed the medications and George did that too. | Did the hospital order the supplies? | No |
| OR | Non-PAR | DEP | The hospital needed the medications that George ordered afterward. | * |  |
| OR | Non-PAR | Control | The hospital needed the medications and George ordered them afterward. | * |  |
| RNR | Non-PAR | DEP | The hospital needed and George ordered the supplies. | Did George need supplies? | No |
| RNR | Non-PAR | Control | The hospital needed the medications and George ordered the supplies. | * |  |
| FILL | Non-PAR | DEP | The hospital was in the suburbs and George did prescriptions. | * |  |
| VPE | PAR | DEP | The soldier protected the communities and the government that temporarily lowered taxes did too. | Did the soldier protect the communities? | Yes |
| VPE | PAR | Control | The soldier protected the communities and the government that temporarily lowered taxes did that too. | * |  |
| OR | PAR | DEP | The soldier protected the communities that the government that temporarily lowered taxes assisted afterward. | * |  |
| OR | PAR | Control | The soldier protected the communities and the government that temporarily lowered taxes assisted them afterward. | Did the government temporarily lowered taxes? | Yes |
| RNR | PAR | DEP | The soldier protected and the government that temporarily lowered taxes assisted the survivors. | * |  |
| RNR | PAR | Control | The soldier protected the communities and the government that temporarily lowered taxes assisted the survivors. | * |  |
| FILL | PAR | DEP | The soldier was in the army and the government that temporarily lowered taxes did recruiting. | Did the soldier do recruiting? | No |
| VPE | Non-PAR | DEP | The soldier protected the communities and Betty did too. | * |  |
| VPE | Non-PAR | Control | The soldier protected the communities and Betty did that too. | * |  |
| OR | Non-PAR | DEP | The soldier protected the communities that Betty assisted afterward. | Did Betty protect the communities? | No |
| OR | Non-PAR | Control | The soldier protected the communities and Betty assisted them afterward. | * |  |
| RNR | Non-PAR | DEP | The soldier protected and Betty assisted the survivors. | * |  |
| RNR | Non-PAR | Control | The soldier protected the communities and Betty assisted the survivors. | Did Betty assist survivors? | Yes |
| FILL | Non-PAR | DEP | The soldier was in the army and Betty did recruiting. | * |  |
| VPE | PAR | DEP | The buyer felt the silks and the tailor who eventually designed clothes did too. | * |  |
| VPE | PAR | Control | The buyer felt the silks and the tailor who eventually designed clothes did that too. | Did the tailor feel the silks? | Yes |
| OR | PAR | DEP | The buyer felt the silks that the tailor who eventually designed clothes measured afterward. | * |  |
| OR | PAR | Control | The buyer felt the silks and the tailor who eventually designed clothes measured them afterward. | * |  |
| RNR | PAR | DEP | The buyer felt and the tailor who eventually designed clothes measured the corduroy. | Did the buyer eventually design clothes? | No |
| RNR | PAR | Control | The buyer felt the silks and the tailor who eventually designed clothes measured the corduroy. | * |  |
| FILL | PAR | DEP | The buyer was in the shop and the tailor who eventually designed clothes did measurements. | * |  |
| VPE | Non-PAR | DEP | The buyer felt the silks and Ryan did too. | Did Ryan measure the silks? | No |
| VPE | Non-PAR | Control | The buyer felt the silks and Ryan did that too. | * |  |
| OR | Non-PAR | DEP | The buyer felt the silks that Ryan measured afterward. | * |  |
| OR | Non-PAR | Control | The buyer felt the silks and Ryan measured them afterward. | Did the buyer feel the silks? | Yes |
| RNR | Non-PAR | DEP | The buyer felt and Ryan measured the corduroy. | * |  |
| RNR | Non-PAR | Control | The buyer felt the silks and Ryan measured the corduroy. | * |  |
| FILL | Non-PAR | DEP | The buyer was in the shop and Ryan did measurements. | Did Ryan do measurements? | Yes |
| VPE | PAR | DEP | The plaintiff blamed the defendants and the judge who permanently disbarred lawyers did too. | * |  |
| VPE | PAR | Control | The plaintiff blamed the defendants and the judge who permanently disbarred lawyers did that too. | * |  |
| OR | PAR | DEP | The plaintiff blamed the defendants that the judge who permanently disbarred lawyers sentenced afterward. | Did the judge blame the defendants? | No |
| OR | PAR | Control | The plaintiff blamed the defendants and the judge who permanently disbarred lawyers sentenced them afterward. | * |  |
| RNR | PAR | DEP | The plaintiff blamed and the judge who permanently disbarred lawyers sentenced the criminals. | * |  |
| RNR | PAR | Control | The plaintiff blamed the defendants and the judge who permanently disbarred lawyers sentenced the criminals. | Did the plaintiff sentence criminals? | No |
| FILL | PAR | DEP | The plaintiff was in the court and the judge who permanently disbarred lawyers did investigations. | * |  |
| VPE | Non-PAR | DEP | The plaintiff blamed the defendants and Ellen did too. | * |  |
| VPE | Non-PAR | Control | The plaintiff blamed the defendants and Ellen did that too. | Did the plaintiff blame the defendants? | Yes |
| OR | Non-PAR | DEP | The plaintiff blamed the defendants that Ellen sentenced afterward. | * |  |
| OR | Non-PAR | Control | The plaintiff blamed the defendants and Ellen sentenced them afterward. | * |  |
| RNR | Non-PAR | DEP | The plaintiff blamed and Ellen sentenced the criminals. | Did Ellen sentence criminals? | Yes |
| RNR | Non-PAR | Control | The plaintiff blamed the defendants and Ellen sentenced the criminals. | * |  |
| FILL | Non-PAR | DEP | The plaintiff was in the court and Ellen did investigations. | * |  |
| VPE | PAR | DEP | The tutor recited the questions and the instructor who reiteratively quizzed teens did too. | Did the tutor reiteratively quiz teens? | No |
| VPE | PAR | Control | The tutor recited the questions and the instructor who reiteratively quizzed teens did that too. | * |  |
| OR | PAR | DEP | The tutor recited the questions that the instructor who reiteratively quizzed teens evaluated afterward. | * |  |
| OR | PAR | Control | The tutor recited the questions and the instructor who reiteratively quizzed teens evaluated them afterward. | Did the tutor evaluate the questions? | No |
| RNR | PAR | DEP | The tutor recited and the instructor who reiteratively quizzed teens evaluated the essays. | * |  |
| RNR | PAR | Control | The tutor recited the questions and the instructor who reiteratively quizzed teens evaluated the essays. | * |  |
| FILL | PAR | DEP | The tutor was in the classroom and the instructor who reiteratively quizzed teens did evaluations. | Did the instructor do evaluations? | Yes |
| VPE | Non-PAR | DEP | The tutor recited the questions and Danny did too. | * |  |
| VPE | Non-PAR | Control | The tutor recited the questions and Danny did that too. | * |  |
| OR | Non-PAR | DEP | The tutor recited the questions that Danny evaluated afterward. | Did the tutor recite the questions? | Yes |
| OR | Non-PAR | Control | The tutor recited the questions and Danny evaluated them afterward. | * |  |
| RNR | Non-PAR | DEP | The tutor recited and Danny evaluated the essays. | * |  |
| RNR | Non-PAR | Control | The tutor recited the questions and Danny evaluated the essays. | Did Danny recite the questions? | No |
| FILL | Non-PAR | DEP | The tutor was in the classroom and Danny did evaluations. | * |  |
| VPE | PAR | DEP | The hotel reimbursed the travelers and the airline that publicly admitted liability did too. | * |  |
| VPE | PAR | Control | The hotel reimbursed the travelers and the airline that publicly admitted liability did that too. | Did the hotel publicly admit liability? | No |
| OR | PAR | DEP | The hotel reimbursed the travelers that the airline that publicly admitted liability delayed afterward. | * |  |
| OR | PAR | Control | The hotel reimbursed the travelers and the airline that publicly admitted liability delayed them afterward. | * |  |
| RNR | PAR | DEP | The hotel reimbursed and the airline that publicly admitted liability delayed the passengers. | Did the hotel reimburse passengers? | Yes |
| RNR | PAR | Control | The hotel reimbursed the travelers and the airline that publicly admitted liability delayed the passengers. | * |  |
| FILL | PAR | DEP | The hotel was in the city and the airline that publicly admitted liability did screenings. | * |  |
| VPE | Non-PAR | DEP | The hotel reimbursed the travelers and Susan did too. | Did the hotel reimburse the travelers? | Yes |
| VPE | Non-PAR | Control | The hotel reimbursed the travelers and Susan did that too. | * |  |
| OR | Non-PAR | DEP | The hotel reimbursed the travelers that Susan delayed afterward. | * |  |
| OR | Non-PAR | Control | The hotel reimbursed the travelers and Susan delayed them afterward. | Did Susan reimburse the travelers? | No |
| RNR | Non-PAR | DEP | The hotel reimbursed and Susan delayed the passengers. | * |  |
| RNR | Non-PAR | Control | The hotel reimbursed the travelers and Susan delayed the passengers. | * |  |
| FILL | Non-PAR | DEP | The hotel was in the city and Susan did screenings. | Did the hotel do screenings? | No |
| VPE | PAR | DEP | The girl pet the puppies and the boy who hardly contained himself did too. | * |  |
| VPE | PAR | Control | The girl pet the puppies and the boy who hardly contained himself did that too. | * |  |
| OR | PAR | DEP | The girl pet the puppies that the boy who hardly contained himself fed afterward. | Did the girl pet the puppies? | Yes |
| OR | PAR | Control | The girl pet the puppies and the boy who hardly contained himself fed them afterward. | * |  |
| RNR | PAR | DEP | The girl pet and the boy who hardly contained himself fed the kittens. | * |  |
| RNR | PAR | Control | The girl pet the puppies and the boy who hardly contained himself fed the kittens. | Did the boy hardly contain himself? | Yes |
| FILL | PAR | DEP | The girl was in the market and the boy who hardly contained himself did shopping. | * |  |
| VPE | Non-PAR | DEP | The girl pet the puppies and Manny did too. | * |  |
| VPE | Non-PAR | Control | The girl pet the puppies and Manny did that too. | Did Manny feed the kittens? | No |
| OR | Non-PAR | DEP | The girl pet the puppies that Manny fed afterward. | * |  |
| OR | Non-PAR | Control | The girl pet the puppies and Manny fed them afterward. | * |  |
| RNR | Non-PAR | DEP | The girl pet and Manny fed the kittens. | Did the girl feed kittens? | No |
| RNR | Non-PAR | Control | The girl pet the puppies and Manny fed the kittens. | * |  |
| FILL | Non-PAR | DEP | The girl was in the market and Manny did shopping. | * |  |
| VPE | PAR | DEP | The butler ignored the doorbells and the maid who chiefly tidied up did too. | Did the maid ignore the doorbells? | Yes |
| VPE | PAR | Control | The butler ignored the doorbells and the maid who chiefly tidied up did that too. | * |  |
| OR | PAR | DEP | The butler ignored the doorbells that the maid who chiefly tidied up answered afterward. | * |  |
| OR | PAR | Control | The butler ignored the doorbells and the maid who chiefly tidied up answered them afterward. | Did the butler ignore the doorbells? | Yes |
| RNR | PAR | DEP | The butler ignored and the maid who chiefly tidied up answered the phones. | * |  |
| RNR | PAR | Control | The butler ignored the doorbells and the maid who chiefly tidied up answered the phones. | * |  |
| FILL | PAR | DEP | The butler was in the foyer and the maid who chiefly tidied up did dusting. | Did the butler chiefly tidied up? | No |
| VPE | Non-PAR | DEP | The butler ignored the doorbells and Millie did too. | * |  |
| VPE | Non-PAR | Control | The butler ignored the doorbells and Millie did that too. | * |  |
| OR | Non-PAR | DEP | The butler ignored the doorbells that Millie answered afterward. | Did Millie ignore doorbells? | No |
| OR | Non-PAR | Control | The butler ignored the doorbells and Millie answered them afterward. | * |  |
| RNR | Non-PAR | DEP | The butler ignored and Millie answered the phones. | * |  |
| RNR | Non-PAR | Control | The butler ignored the doorbells and Millie answered the phones. | Did Millie answer phones? | Yes |
| FILL | Non-PAR | DEP | The butler was in the foyer and Millie did dusting. | * |  |
| VPE | PAR | DEP | The mover loaded the boxes and the crew who randomly showed up did too. | * |  |
| VPE | PAR | Control | The mover loaded the boxes and the crew who randomly showed up did that too. | Did the mover load the boxes? | Yes |
| OR | PAR | DEP | The mover loaded the boxes that the crew who randomly showed up unloaded afterward. | * |  |
| OR | PAR | Control | The mover loaded the boxes and the crew who randomly showed up unloaded them afterward. | * |  |
| RNR | PAR | DEP | The mover loaded and the crew who randomly showed up unloaded the furniture. | Did the mover unload furniture? | No |
| RNR | PAR | Control | The mover loaded the boxes and the crew who randomly showed up unloaded the furniture. | * |  |
| FILL | PAR | DEP | The mover was in the truck and the crew who randomly showed up did packing. | * |  |
| VPE | Non-PAR | DEP | The mover loaded the boxes and Richard did too. | Did Richard unload furniture? | No |
| VPE | Non-PAR | Control | The mover loaded the boxes and Richard did that too. | * |  |
| OR | Non-PAR | DEP | The mover loaded the boxes that Richard unloaded afterward. | * |  |
| OR | Non-PAR | Control | The mover loaded the boxes and Richard unloaded them afterward. | Did Richard unload the boxes? | Yes |
| RNR | Non-PAR | DEP | The mover loaded and Richard unloaded the furniture. | * |  |
| RNR | Non-PAR | Control | The mover loaded the boxes and Richard unloaded the furniture. | * |  |
| FILL | Non-PAR | DEP | The mover was in the truck and Richard did packing. | Did Richard do packing? | Yes |
| VPE | PAR | DEP | The critic tasted the wines and the customer who sparingly purchased alcohol did too. | * |  |
| VPE | PAR | Control | The critic tasted the wines and the customer who sparingly purchased alcohol did that too. | * |  |
| OR | PAR | DEP | The critic tasted the wines that the customer who sparingly purchased alcohol selected afterward. | Did the critic sparingly purchased alcohol? | No |
| OR | PAR | Control | The critic tasted the wines and the customer who sparingly purchased alcohol selected them afterward. | * |  |
| RNR | PAR | DEP | The critic tasted and the customer who sparingly purchased alcohol selected the cheese. | * |  |
| RNR | PAR | Control | The critic tasted the wines and the customer who sparingly purchased alcohol selected the cheese. | Did the critic select cheese? | No |
| FILL | PAR | DEP | The critic was in the winery and the customer who sparingly purchased alcohol did tastings. | * |  |
| VPE | Non-PAR | DEP | The critic tasted the wines and Mindy did too. | * |  |
| VPE | Non-PAR | Control | The critic tasted the wines and Mindy did that too. | Did Mindy taste the wines? | Yes |
| OR | Non-PAR | DEP | The critic tasted the wines that Mindy selected afterward. | * |  |
| OR | Non-PAR | Control | The critic tasted the wines and Mindy selected them afterward. | * |  |
| RNR | Non-PAR | DEP | The critic tasted and Mindy selected the cheese. | Did the critic taste cheese? | Yes |
| RNR | Non-PAR | Control | The critic tasted the wines and Mindy selected the cheese. | * |  |
| FILL | Non-PAR | DEP | The critic was in the winery and Mindy did tastings. | * |  |
| VPE | PAR | DEP | The couple acknowledged the donors and the kids who quietly played together did too. | Did the couple thank the donors? | No |
| VPE | PAR | Control | The couple acknowledged the donors and the kids who quietly played together did that too. | * |  |
| OR | PAR | DEP | The couple acknowledged the donors that the kids who quietly played together thanked afterward. | * |  |
| OR | PAR | Control | The couple acknowledged the donors and the kids who quietly played together thanked them afterward. | Did the couple quietly play together? | No |
| RNR | PAR | DEP | The couple acknowledged and the kids who quietly played together thanked the helpers. | * |  |
| RNR | PAR | Control | The couple acknowledged the donors and the kids who quietly played together thanked the helpers. | * |  |
| FILL | PAR | DEP | The couple was in the neighborhood and the kids who quietly played together did activities. | Did the kids do activities? | Yes |
| VPE | Non-PAR | DEP | The couple acknowledged the donors and Morgan did too. | * |  |
| VPE | Non-PAR | Control | The couple acknowledged the donors and Morgan did that too. | * |  |
| OR | Non-PAR | DEP | The couple acknowledged the donors that Morgan thanked afterward. | Did Morgan thank the donors? | Yes |
| OR | Non-PAR | Control | The couple acknowledged the donors and Morgan thanked them afterward. | * |  |
| RNR | Non-PAR | DEP | The couple acknowledged and Morgan thanked the helpers. | * |  |
| RNR | Non-PAR | Control | The couple acknowledged the donors and Morgan thanked the helpers. | Did the couple thank helpers? | No |
| FILL | Non-PAR | DEP | The couple was in the neighborhood and Morgan did activities. | * |  |
| VPE | PAR | DEP | The priest uttered the prayers and the nun who stoically confessed sins did too. | * |  |
| VPE | PAR | Control | The priest uttered the prayers and the nun who stoically confessed sins did that too. | Did the nun chant the prayers? | No |
| OR | PAR | DEP | The priest uttered the prayers that the nun who stoically confessed sins chanted afterward. | * |  |
| OR | PAR | Control | The priest uttered the prayers and the nun who stoically confessed sins chanted them afterward. | * |  |
| RNR | PAR | DEP | The priest uttered and the nun who stoically confessed sins chanted the scripture. | Did the nun stoically confess sins? | Yes |
| RNR | PAR | Control | The priest uttered the prayers and the nun who stoically confessed sins chanted the scripture. | * |  |
| FILL | PAR | DEP | The priest was in the church and the nun who stoically confessed sins did communion. | * |  |
| VPE | Non-PAR | DEP | The priest uttered the prayers and Gwen did too. | Did the priest utter the prayers? | Yes |
| VPE | Non-PAR | Control | The priest uttered the prayers and Gwen did that too. | * |  |
| OR | Non-PAR | DEP | The priest uttered the prayers that Gwen chanted afterward. | * |  |
| OR | Non-PAR | Control | The priest uttered the prayers and Gwen chanted them afterward. | Did Gwen utter the prayers? | No |
| RNR | Non-PAR | DEP | The priest uttered and Gwen chanted the scripture. | * |  |
| RNR | Non-PAR | Control | The priest uttered the prayers and Gwen chanted the scripture. | * |  |
| FILL | Non-PAR | DEP | The priest was in the church and Gwen did communion. | Did the priest do communion? | No |
| VPE | PAR | DEP | The counselor comforted the victims and the family who kindly offered help did too. | * |  |
| VPE | PAR | Control | The counselor comforted the victims and the family who kindly offered help did that too. | * |  |
| OR | PAR | DEP | The counselor comforted the victims that the family who kindly offered help hugged afterward. | Did the family hug the victims? | Yes |
| OR | PAR | Control | The counselor comforted the victims and the family who kindly offered help hugged them afterward. | * |  |
| RNR | PAR | DEP | The counselor comforted and the family who kindly offered help hugged the children. | * |  |
| RNR | PAR | Control | The counselor comforted the victims and the family who kindly offered help hugged the children. | Did the counselor comfort the victims? | Yes |
| FILL | PAR | DEP | The counselor was in the clinic and the family who kindly offered help did therapy. | * |  |
| VPE | Non-PAR | DEP | The counselor comforted the victims and Earl did too. | * |  |
| VPE | Non-PAR | Control | The counselor comforted the victims and Earl did that too. | Did Earl hug the children? | No |
| OR | Non-PAR | DEP | The counselor comforted the victims that Earl hugged afterward. | * |  |
| OR | Non-PAR | Control | The counselor comforted the victims and Earl hugged them afterward. | * |  |
| RNR | Non-PAR | DEP | The counselor comforted and Earl hugged the children. | Did the counselor hug children? | No |
| RNR | Non-PAR | Control | The counselor comforted the victims and Earl hugged the children. | * |  |
| FILL | Non-PAR | DEP | The counselor was in the clinic and Earl did therapy. | * |  |
| VPE | PAR | DEP | The newspaper showed the photos and the newscaster who vividly described events did too. | Did the newscaster vividly describe events? | Yes |
| VPE | PAR | Control | The newspaper showed the photos and the newscaster who vividly described events did that too. | * |  |
| OR | PAR | DEP | The newspaper showed the photos that the newscaster who vividly described events presented afterward. | * |  |
| OR | PAR | Control | The newspaper showed the photos and the newscaster who vividly described events presented them afterward. | Did the newspaper show the photos? | Yes |
| RNR | PAR | DEP | The newspaper showed and the newscaster who vividly described events presented the headlines. | * |  |
| RNR | PAR | Control | The newspaper showed the photos and the newscaster who vividly described events presented the headlines. | * |  |
| FILL | PAR | DEP | The newspaper was in the studio and the newscaster who vividly described events did coverage. | Did the newspaper do coverage? | No |
| VPE | Non-PAR | DEP | The newspaper showed the photos and Brenda did too. | * |  |
| VPE | Non-PAR | Control | The newspaper showed the photos and Brenda did that too. | * |  |
| OR | Non-PAR | DEP | The newspaper showed the photos that Brenda presented afterward. | Did Brenda do coverage? | No |
| OR | Non-PAR | Control | The newspaper showed the photos and Brenda presented them afterward. | * |  |
| RNR | Non-PAR | DEP | The newspaper showed and Brenda presented the headlines. | * |  |
| RNR | Non-PAR | Control | The newspaper showed the photos and Brenda presented the headlines. | Did Brenda present headlines? | Yes |
| FILL | Non-PAR | DEP | The newspaper was in the studio and Brenda did coverage. | * |  |
| VPE | PAR | DEP | The archaeologist probed the artifacts and the scholar who diligently scribbled notes did too. | * |  |
| VPE | PAR | Control | The archaeologist probed the artifacts and the scholar who diligently scribbled notes did that too. | Did the scholar diligently scribble notes? | Yes |
| OR | PAR | DEP | The archaeologist probed the artifacts that the scholar who diligently scribbled notes authenticated afterward. | * |  |
| OR | PAR | Control | The archaeologist probed the artifacts and the scholar who diligently scribbled notes authenticated them afterward. | * |  |
| RNR | PAR | DEP | The archaeologist probed and the scholar who diligently scribbled notes authenticated the letters. | Did the scholar probe the artifacts? | No |
| RNR | PAR | Control | The archaeologist probed the artifacts and the scholar who diligently scribbled notes authenticated the letters. | * |  |
| FILL | PAR | DEP | The archaeologist was in the ruins and the scholar who diligently scribbled notes did fieldwork. | * |  |
| VPE | Non-PAR | DEP | The archaeologist probed the artifacts and Marty did too. | Did the archaeologist authenticate letters? | No |
| VPE | Non-PAR | Control | The archaeologist probed the artifacts and Marty did that too. | * |  |
| OR | Non-PAR | DEP | The archaeologist probed the artifacts that Marty authenticated afterward. | * |  |
| OR | Non-PAR | Control | The archaeologist probed the artifacts and Marty authenticated them afterward. | Did Marty authenticate the artifacts? | Yes |
| RNR | Non-PAR | DEP | The archaeologist probed and Marty authenticated the letters. | * |  |
| RNR | Non-PAR | Control | The archaeologist probed the artifacts and Marty authenticated the letters. | * |  |
| FILL | Non-PAR | DEP | The archaeologist was in the ruins and Marty did fieldwork. | Did Marty do fieldwork? | Yes |
| VPE | PAR | DEP | The witness described the events and the reporter who deliberately avoided scandal did too. | * |  |
| VPE | PAR | Control | The witness described the events and the reporter who deliberately avoided scandal did that too. | * |  |
| OR | PAR | DEP | The witness described the events that the reporter who deliberately avoided scandal recounted afterward. | Did the reporter describe the events? | No |
| OR | PAR | Control | The witness described the events and the reporter who deliberately avoided scandal recounted them afterward. | * |  |
| RNR | PAR | DEP | The witness described and the reporter who deliberately avoided scandal recounted the details. | * |  |
| RNR | PAR | Control | The witness described the events and the reporter who deliberately avoided scandal recounted the details. | Did the witness deliberately avoid scandal? | No |
| FILL | PAR | DEP | The witness was in the precinct and the reporter who deliberately avoided scandal did nothing. | * |  |
| VPE | Non-PAR | DEP | The witness described the events and Leah did too. | * |  |
| VPE | Non-PAR | Control | The witness described the events and Leah did that too. | Did Leah describe the events? | Yes |
| OR | Non-PAR | DEP | The witness described the events that Leah recounted afterward. | * |  |
| OR | Non-PAR | Control | The witness described the events and Leah recounted them afterward. | * |  |
| RNR | Non-PAR | DEP | The witness described and Leah recounted the details. | Did the witness describe details? | Yes |
| RNR | Non-PAR | Control | The witness described the events and Leah recounted the details. | * |  |
| FILL | Non-PAR | DEP | The witness was in the precinct and Leah did nothing. | * |  |
| VPE | PAR | DEP | The user accessed the websites and the programmers who carefully designed software did too. | Did the user update the websites? | No |
| VPE | PAR | Control | The user accessed the websites and the programmers who carefully designed software did that too. | * |  |
| OR | PAR | DEP | The user accessed the websites that the programmers who carefully designed software updated afterward. | * |  |
| OR | PAR | Control | The user accessed the websites and the programmers who carefully designed software updated them afterward. | Did the programmers access the websites? | No |
| RNR | PAR | DEP | The user accessed and the programmers who carefully designed software updated the files. | * |  |
| RNR | PAR | Control | The user accessed the websites and the programmers who carefully designed software updated the files. | * |  |
| FILL | PAR | DEP | The user was in the library and the programmers who carefully designed software did testing. | Did the programmers carefully design software? | Yes |
| VPE | Non-PAR | DEP | The user accessed the websites and Chris did too. | * |  |
| VPE | Non-PAR | Control | The user accessed the websites and Chris did that too. | * |  |
| OR | Non-PAR | DEP | The user accessed the websites that Chris updated afterward. | Did the user access the websites? | Yes |
| OR | Non-PAR | Control | The user accessed the websites and Chris updated them afterward. | * |  |
| RNR | Non-PAR | DEP | The user accessed and Chris updated the files. | * |  |
| RNR | Non-PAR | Control | The user accessed the websites and Chris updated the files. | Did the user update files? | No |
| FILL | Non-PAR | DEP | The user was in the library and Chris did testing. | * |  |
| VPE | PAR | DEP | The campaigners pronounced the slogans and the rioters who destructively smashed windows did too. | * |  |
| VPE | PAR | Control | The campaigners pronounced the slogans and the rioters who destructively smashed windows did that too. | Did the rioters do marches? | No |
| OR | PAR | DEP | The campaigners pronounced the slogans that the rioters who destructively smashed windows repeated afterward. | * |  |
| OR | PAR | Control | The campaigners pronounced the slogans and the rioters who destructively smashed windows repeated them afterward. | * |  |
| RNR | PAR | DEP | The campaigners pronounced and the rioters who destructively smashed windows repeated the chants. | Did the campaigners pronounce chants? | Yes |
| RNR | PAR | Control | The campaigners pronounced the slogans and the rioters who destructively smashed windows repeated the chants. | * |  |
| FILL | PAR | DEP | The campaigners were in the street and the rioters who destructively smashed windows did marches. | * |  |
| VPE | Non-PAR | DEP | The campaigners pronounced the slogans and Samantha did too. | Did the rioters pronounce slogans? | Yes |
| VPE | Non-PAR | Control | The campaigners pronounced the slogans and Samantha did that too. | * |  |
| OR | Non-PAR | DEP | The campaigners pronounced the slogans that Samantha repeated afterward. | * |  |
| OR | Non-PAR | Control | The campaigners pronounced the slogans and Samantha repeated them afterward. | Did the campaigners repeat the slogans? | No |
| RNR | Non-PAR | DEP | The campaigners pronounced and Samantha repeated the chants. | * |  |
| RNR | Non-PAR | Control | The campaigners pronounced the slogans and Samantha repeated the chants. | * |  |
| FILL | Non-PAR | DEP | The campaigners were in the street and Samantha did marches. | Did Samantha repeat the slogans? | No |
